# A novel RNA aptamer identifies plasma membrane ATP synthase beta subunit as an early marker and therapeutic target in aggressive cancer

**DOI:** 10.1007/s10549-019-05174-3

**Published:** 2019-04-20

**Authors:** S. Speransky, P. Serafini, J. Caroli, S. Bicciato, M. E. Lippman, N. H. Bishopric

**Affiliations:** 1 Department of Medicine, Sylvester Comprehensive Cancer Center, University of Miami Miller School of Medicine, Miami, USA; 2 Department of Microbiology & Immunology, Sylvester Comprehensive Cancer Center, University of Miami Miller School of Medicine, Miami, USA; 3 Center for Genome Research, Department of Life Sciences, University of Modena and Reggio Emilia, Modena, Italy; 4 Department of Oncology, Georgetown Lombardi Comprehensive Cancer Center, Georgetown University, Washington, DC USA

**Keywords:** Ecto-ATP synthase, Metastasis, Breast cancer, Prostate cancer, Aptamer, Cell-SELEX

## Abstract

**Purpose:**

Primary breast and prostate cancers can be cured, but metastatic disease cannot. Identifying cell factors that predict metastatic potential could guide both prognosis and treatment.

**Methods:**

We used Cell-SELEX to screen an RNA aptamer library for differential binding to prostate cancer cell lines with high vs. low metastatic potential. Mass spectroscopy, immunoblot, and immunohistochemistry were used to identify and validate aptamer targets. Aptamer properties were tested in vitro, in xenograft models, and in clinical biopsies. Gene expression datasets were queried for target associations in cancer.

**Results:**

We identified a novel aptamer (Apt63) that binds to the beta subunit of F_1_F_o_ ATP synthase (ATP5B), present on the plasma membrane of certain normal and cancer cells. Apt63 bound to plasma membranes of multiple aggressive breast and prostate cell lines, but not to normal breast and prostate epithelial cells, and weakly or not at all to non-metastasizing cancer cells; binding led to rapid cell death. A single intravenous injection of Apt63 induced rapid, tumor cell-selective binding and cytotoxicity in MDA-MB-231 xenograft tumors, associated with endonuclease G nuclear translocation and DNA fragmentation. Apt63 was not toxic to non-transformed epithelial cells in vitro or adjacent normal tissue in vivo. In breast cancer tissue arrays, plasma membrane staining with Apt63 correlated with tumor stage (*p* < 0.0001, *n* = 416) and was independent of other cancer markers. Across multiple datasets, ATP5B expression was significantly increased relative to normal tissue, and negatively correlated with metastasis-free (*p* = 0.0063, 0.00039, respectively) and overall (*p* = 0.050, 0.0198) survival.

**Conclusion:**

Ecto-ATP5B binding by Apt63 may disrupt an essential survival mechanism in a subset of tumors with high metastatic potential, and defines a novel category of cancers with potential vulnerability to ATP5B-targeted therapy. Apt63 is a unique tool for elucidating the function of surface ATP synthase, and potentially for predicting and treating metastatic breast and prostate cancer.

**Electronic supplementary material:**

The online version of this article (10.1007/s10549-019-05174-3) contains supplementary material, which is available to authorized users.

## Introduction

Localized prostate and breast cancers are highly curable, but once metastasized to remote organs, these cancers are inevitably lethal. Consequently, an important goal of treatment is to identify and exploit specific vulnerabilities of the metastatic cell. Another key aim is to predict which tumors are at high risk of metastasis, allowing potentially toxic therapy to be tailored to those most likely to benefit. These goals have been aided by an improved understanding of cancer cell genetic drift during tumor progression, which allows certain cells to acquire independence from supportive factors in the tissue of origin, to migrate into the vasculature, and to survive and grow at foreign sites such as liver and bone. In addition to genetic drivers of the metastatic phenotype [1–5], epigenetic and protein-level changes have been found to establish tumor cell aggressiveness, including episomal transfer of microRNAs to and from adjacent normal cells, reprogramming of the tumor or stroma by factors released from tumor-infiltrating lymphocytes, and alterations in metabolism brought about by tumor hypoxia [6–8]. Energy production from carbon sources is frequently deranged in cancer, and may be associated with changes in the epigenetic state of the cell that promote cell-autonomous increases in tumor aggression (reviewed in [8]). A thorough search for metastasis-promoting changes in the cancer cell thus necessarily extends to exploration of protein content, function, and location.

In this study, we used an unsupervised method: differential Cell-SELEX (Systematic Evolution of Ligands by EXponential enrichment), to search for proteins distinguishing metastatic from non-metastatic subclones of a single parental prostate cancer cell line, LNCaP. Owing to their unique, sequence-specific tertiary structure, single-stranded nucleic acids (either DNA or RNA) can bind to individual proteins with high specificity and affinity, comparable to those of antibodies. These oligonucleotides, known as aptamers, can be modified for stability in biological fluids, labeled with fluorescent tags, fused to other molecules, and delivered in vivo without inciting an immune response [9]. Cell-SELEX uses live cells to select aptamers that recognize cellular proteins in their native and functional state. Differential Cell-SELEX applies the same method to identify aptamers that discriminate between two cell types. The ability to screen large numbers (> 2^40^) of sequences increases the likelihood of identifying rare or unique surface marker differences.

Here, we report the identification of a novel RNA aptamer (Apt63) that recognizes a plasma membrane feature that is commonly expressed by multiple aggressive prostate and breast cancer cell lines and tumors, but that exhibits low expression or is absent in non-transformed cells and normal tissues. We demonstrate that the aptamer target is the beta subunit of F_1_F_o_ ATP synthase (ATP5B). This protein is a catalytic component of the final enzyme in cellular ATP production by oxidative phosphorylation, and is located on the inner mitochondrial membrane. ATP5B and other components of the F_1_F_o_ ATP synthase complex have previously been identified on the plasma membrane of certain cell and tumor types, where it is referred to as “ecto-ATP synthase”; several studies have shown that the complex is catalytically active in extracellular ATP production [10, 11]. Various roles have been established for this activity in a few normal cell types, and particularly in angiogenesis, but its significance and function in cancer remain uncertain. Ecto-ATP synthase acts as a ligand for angiostatin and transduces some of its anti-proliferative and anti-angiogenic effects [12]. Binding to ecto-ATP synthase by angiostatin, membrane-impermeable small molecules and monoclonal antibodies against the ATP5 beta subunit have been shown to promote cell death in a wide range of susceptible cell types, including HeLa, Leishmania, and plant cells ([13] and citations therein; [14–16]). Several studies have linked expression of surface ATP synthase to more-aggressive and later-stage cancer [17, 18], suggesting that the activity of this complex on the cell surface may support the survival of these aggressive cells during the transition to metastasis. In this study, we show that Apt63 distinguishes aggressive breast and prostate cancer cell lines from less-aggressive congenic lines, and from non-transformed cells, both human and murine. In vivo, Apt63 binds selectively to ecto-ATP5B-expressing tumors and not to normal adjacent tissue. Functionally, binding of Apt63 to the plasma membrane exerts selective tumor cell killing by inducing translocation of endonuclease G from mitochondria to nucleus, DNA fragmentation, and apoptosis. We show that that Apt63 plasma membrane binding in clinical tissue biopsies is strongly correlated with advanced tumor stage, and as a corollary, that ATP5B expression in primary tumors is predictive of poor metastasis-free and overall survival. We propose that Apt63 may be useful in early recognition and treatment of a novel subset of highly aggressive primary breast and prostate cancers, defined by surface expression of ATP5B.

## Materials and methods

### Cell lines and cell culture

Human prostate cancer cell lines used in the Cell-SELEX screen were obtained from Dr. Curtis Pettaway [19]. Human prostate cancer cells (PC-3, PC3-ML, RWPE-1) were generously provided by Dr. Kerry Burnstein (University of Miami)^,^ and human breast cancer cell lines (MDA-MB-231, MDA-MB-436, MCF7, MCF10) were obtained from ATCC (Manassas, VA), Murine breast cancer cells lines (4T1, 67NR, E0771, E0771.LMB) were the gift of Dr. Barry Hudson (University of Miami). Dissociated primary tumor lines DT28 and DT22 were the generous gift of Dr. D. El-Ashry (University of Minnesota) [20]. All cell lines were maintained using the suppliers’ protocols and maintained in 37 °C, 5% CO_2_ tissue culture incubators. All cell lines were routinely tested for mycoplasma using the MycoAlert Mycoplasma Detection Kit (Lonza, Walkersville, MD, USA) and an established PCR protocol [21].

### Differential Cell-SELEX

The pool of RNA aptamers used for Cell-SELEX was obtained from a cDNA library with the general template: TCT CGG ATC CTC AGC GAG TCG TCT G-(N40)-CCG CAT CGT CCT CCC TA (where N40 represents 40 random nucleotides). The cDNA library was amplified by PCR and transcribed in vitro using a Durascribe T7 RNA synthesis kit (Lucigene, USA) with nuclease-stable 2ʹ-F-dCTP and 2ʹ-F-dUTP as previously described [22]. The aptamer library was purified using an RNeasy kit (Qiagen). Both parental and LNCaP-Pro5 (Pro5) cells were used for negative selection, and LNCaP-LN3 (LN3) for positive selection. Each Cell-SELEX cycle consists two rounds of selection, negative and positive. Parental LNCaP cells were used in the first three selection cycles and Pro5 cells in cycles 4–11. At the beginning of each cycle, 1 µg of the aptamer library was added to 450 µL of PBS containing 0.5 mM MgCl2 and 1 mM CaCl_2_ (binding buffer) and RNA was refolded by heating 5 min at 67 °C and cooling at room temperature (RT) for 10 min. All cells used in Cell-SELEX were grown in 75T culture flasks with filtered cups (ThermoFisher Scientific). At 75% confluency, cells were briefly washed with PBS and dissociated from the flask by incubation with 3 ml of Trypsin–EDTA (0.25%) without phenol red (ThermoFisher Scientific) in RT for 3 min. 10 ml of growing media was added to halt Trypsin–EDTA reaction. Detached cells were collected into 15 ml conical tubes (Falcon) and centrifuged for 5 min in a 4 °C tabletop centrifuge (Eppendorf) at 600×*g*. Cell pellets were resuspended in 5 ml of binding buffer and counted. 2 × 10^5^ Pro5 cells were separated into fresh tubes and centrifuged in tabletop centrifuge for 5 min at RT; the cell pellet was then resuspended with the aptamer library and incubated for 10 min at RT on a circular rotator to continuously agitate the cells. Cells were again centrifuged for 5 min at RT, and the supernatant, containing aptamers not bound to the negative selector, was collected and filtered by passing through 0.2 µm Pall Acrodisc® Sterile Syringe Filters with Supor® Membrane (Pall Laboratory). In the positive selection step, 0.5 × 10^5^ LN3 cells were first incubated for 10 min at RT with 0.1 mg/ml of yeast tRNA (Sigma) to reduce non-specific RNA binding, then LN3 cells were washed with binding buffer, centrifuged, and the cell pellet resuspended with the filtered supernatant from the negative selection. LN3 cells were incubated for 10 min on a rotator at RT, followed by isolation of total RNA (including bound aptamers) using Trizol reagent (Invitrogen). RNA aptamers were transcribed from total RNA using an aptamer-specific forward primer and a SuperScript® III Reverse Transcriptase reaction (Invitrogen), and amplified from first strand cDNA by standard PCR (95 °C, 5′, 3 × (94 °C 30″, 52 °C 20″, 72 °C 25″), 15 × (94 °C 30″, 54 °C 20″, 72 °C 25″), 72 °C 5). RNA sequences were transcribed from the resulting cDNA pool using a Durascribe T7 kit as detailed above, and entered into the next Cell-SELEX cycle. RNA aptamer pools were sampled at cycles 1, 4, and 11. After cycle 11, aptamer pools were sequenced, aligned, and analyzed to select candidates for further study as previously described [22, 23].

### Generation of aptamers and scrambled sequences for in vitro and in vivo experiments

Selected aptamers and scrambled oligomers were synthesized by Integrated DNA Technologies (IDT, USA) and used as templates for amplification by PCR (95 °C, 5′, 3 × (94 °C 30″, 52 °C 20″, 72 °C 25″), 15 × (94 °C 30″, 54 °C 20″, 72 °C 25″), 72 °C 5′) with the universal forward primer 5′-GGG GGA ATT CTA ATA CGA CTC ACT ATA GGG AGG ACG ATG CGG-3, and the reverse primer 5′-TCT CGG ATC CTC AGC GAG TCG TC-3′ (oligomer sequences are listed in Online Resource 1). RNA sequences were then transcribed by Durascribe T7 kit (Lucigene, USA) following the manufacturer’s protocol. RNA aptamers and scrambled sequences were labeled for in vitro experiments using Silencer™ siRNA Labeling Kit with Cy™3 dye (Thermo Fisher Scientific) and for in vivo experiments using Ulysis Alexa Fluor™ 647 Nucleic Acid Labeling Kit (Thermo Fisher Scientific) following the manufacturer’s recommended protocols.

### Fluorescence microscopy for aptamer imaging

Cells were seeded into 35 mm glass bottom dishes (MatTek Corporation, Ashland, MA) at a density of 0.3 × 10^6^ cells per dish, and allowed to grow for 48 h to 60–75% confluence. Cy3-labeled aptamers were added to culture media at a final concentration of 1 nM and incubated with live cells for 30 min in 37 °C in 5% CO_2_. Following incubation, cells were washed 3 × for 5 min each with PBS and fixed 10 min with 4% paraformaldehyde at RT. After fixation, cells were washed with PBS and counterstained with DAPI (Sigma), 1 µg final concentration for 5 min. To identify membrane co-localization of Cy3-Apt63 and ATP5B antibody, cells were grown on coverslips in 35 mm tissue culture dishes, and stained sequentially with Cy3-Apt63 and AlexaFluor®647 anti-ATP5B antibodies (ab223436, ABCAM), without a permeabilization step. To co-localize Apt63 and ATP5B antibody within mitochondria, cells were treated with 0.05% Triton X-100 for 5 min, washed three times with PBS, then incubated with both the AlexaFluor®647 anti-ATP5B antibody and Cy3-Apt63. Finally, cells were counterstained with DAPI. For some experiments, live cells were first stained with Cy3-Apt63, followed by fixation, treatment with 0.05% Triton X-100, and DAPI counterstaining as described above. Coverslips were mounted with ProLong®Gold antifade reagent (Life Technologies). Fluorescent images were obtained on a confocal microscope (Leica SP5) using a 20x dry objective (Leica PL APO CS).

### Aptamer target purification and identification

Apt63 and scrambled sequence (AptScr) were 3′ end-biotinylated using Pierce™ RNA 3′ End Desthiobiotinylation Kit (ThermoFisher Scientific, USA) following the manufacturer’s protocol. LNCaP-LN3 and LNCaP-Pro5 cells were each seeded into 100 mm Petri dishes for 48 h at a density 0.5 × 10^6^ cells per dish and allowed to grow to 60–75% confluency. On the day of the experiment, cells were incubated at RT for 1 h with the desthiobiotin-RNA-Apt63 or -AptScr complexes, allowing aptamer to bind to target. Following binding, cells were washed 3 × for 5 min each in PBS at RT to remove excess unbound RNA-desthiobiotin complexes, and cross-linked by incubation with 1% paraformaldehyde for 2 min. Next, cells were thoroughly washed 3 × for 5 min each in PBS at RT. To separate membranes from intracellular components, cells were incubated in a mild hypotonic lysis buffer containing 1 M Tris–HCl, 5M NaCl, 50 mM MgCl, 0.1M DTT, and protease inhibitor cocktail for 2 min on ice. Immediately following incubation, cells were gently homogenized in a Dounce homogenizer, ten times on ice, mixed with magnetic beads and left overnight at 4 °C to allow capture of the desthiobiotin-aptamer-target hybrid complexes. On the next day, the beads were thoroughly washed with reagents provided in the kit, and target-aptamer complexes eluted with 30 µL of 8M urea for 10 min at 60 °C. The recovered eluates and total cell lysate were separated on 4–20% gradient SDS–PAGE gels (Bio-Rad, USA). Gels were stained using Pierce™ Silver Stain kits. Protein band distributions were compared between LNCaP-LN3 and LNCaP-Pro5 cell lines, and the most enriched band in the LN3 aptamer-target eluate was cut, sequenced by microcapillary LS/MS/MS, and analyzed by SEQUEST software at Taplin Mass Spectrometry Facility (Harvard Medical School, Boston MA). The predicted protein target was verified by 4–20% gradient SDS-PAGE gel electrophoresis and western blot in whole cell lysates and aptamer eluates using ATP5B antibodies (ab170947, ABCAM) with ATP5B recombinant protein as a positive control (ab92235, ABCAM).

### In vitro aptamer binding affinity and cytotoxicity assays

We used two independent methods to evaluate Apt63 cytotoxicity in a series of cell lines in vitro: (1) direct visualization of Apt63 cytotoxicity using the IncuCyte® S3 Live-Cell Analysis System, and (2) SYTOX™ Green uptake. Binding affinity of Apt63 to its membrane target was measured using the CellTiter-Glo® system (Promega). Detailed procedures are described in Online Resource 2.

### Mouse xenograft models and aptamer cytotoxicity in vivo

All animal experiments were approved by and performed in accordance with the guidelines of the University of Miami Institutional Animal Care and Use Committee. For live visualization of Apt63 tumor uptake and retention in vivo, a prostate xenograft tumor model was used. NOD.CB17-Prkdcscid/J male mice (10 weeks old, *n* = 14, The Jackson Laboratory) were injected orthotopically into the right anterior lobe of the prostate with 2 × 10^6^ LN3 and 2 × 10^6^ Pro5 cells. For Apt63 cytotoxicity in vivo, we used a previously described breast tumor xenograft mouse model [24]. NOD.CB17-Prkdcscid/J female mice (6–8 weeks old, *n* = 39, The Jackson Laboratory) were injected with 10^6^ of MDA-MB-231 cells into the mammary fat pad. In both xenograft models, when tumors were palpable, 1 nmol of Alexa Fluor™ 647-labeled Apt63, AptScr, or unlabeled oligonucleotides suspended in 200 µL of PBS was injected into the tail vein as a single injection. Mice were imaged and euthanized at specified time points. Tumors and selected tissues were dissected and processed for further analysis. Detailed procedures are described in Online Resource 2.

### Tumor tissue and FFPE human biopsy arrays fluorescent staining and analysis

Xenograft tumors were removed from euthanized mice and immediately frozen or fixed with 10% buffered formalin (VWR, USA), paraffin embedded, and processed. Prostate and breast core tissue microarrays (TMA) were purchased from US Biomax, Inc (Rockville, MD). The detailed staining protocols are provided in as described in Online Resource 2. Cy3-Apt63-stained human breast biopsy microarrays were imaged by fluorescence microscopy on a Virtual Slide Microscope (VS120) for overview images and on a confocal microscope (Leica SP5) for high-resolution images, and scored for visual presence or absence (greater or less than 10% of cells, respectively) of Apt63 membrane-specific labeling. A list of TMAs with patient information, tumor grade, and stage used in this study and assigned Apt63 score for each biopsy is provided in Online Resource 3. Statistical analysis was performed for the Pearson correlation coefficient of aptamer membrane-specific stain vs. histopathological grades and stages.

### ATP5B expression datasets and analysis

ATP5B gene expression was analyzed in prostate and breast cancer samples downloaded from Gene Expression Omnibus and from the Genomic Data Commons Portal. Specifically, RNA-seq data in FPKM (Fragments Per Kilobase Million) and clinical information of the TCGA Prostate Adenocarcinoma dataset (TCGA-PRAD [25]) were downloaded from the Genomic Data Commons Portal using functions of the *TCGAbiolinks* R package and used as is. Expression levels of prostate tumors (*n* = 264) and normal prostate tissue samples (*n* = 160) from Penney et al. [26] were downloaded from GEO GSE62872 as Series Matrix File and used as is. Raw data of 545 formalin-fixed paraffin-embedded (FFPE) tissue samples from primary prostate cancer were downloaded from GEO GSE46691 [27]. Probe-level signals were converted to expression values from CEL files using robust multi-array average procedure RMA [28] and an Entrez gene-centered custom CDF for Affymetrix Human Exon 1.0 ST Array (http://brainarray.mbni.med.umich.edu/Brainarray/Database/CustomCDF/CDF_download.asp; version 22). Gene expression profiles of 25 matched normal and tumor breast tissues were downloaded from GEO GSE109169 [29] as Series Matrix File and used as is. Full expression median-centered data, consisting of 522 primary tumors, 3 metastatic tumors, and 22 tumor-adjacent normal samples, and clinical information of the TCGA Breast Invasive Carcinoma dataset (TCGA-BRCA;[30]) were downloaded from https://tcga-data.nci.nih.gov/docs/publications/brca_2012/ and used as is. Finally, we used a breast cancer compendium created from a collection of 4640 samples from 27 major datasets containing microarray data on breast cancer samples annotated with clinical information. The compendium consists of a meta-dataset of gene expression data for 3,661 unique samples from 25 independent cohorts [31, 32].

All data analyses were performed in R (version 3.5.1) using Bioconductor libraries (BioC 3.7) and R statistical packages. To identify two groups of tumors with either high or low ATP5B expression, we used the classifier described in [33], based on the standardized expression (score) of a gene or a signature. Tumors were classified as ATP5B ‘Low’ if the ATP5B score was negative and as ATP5B ‘High’ if the ATP5B score was positive. To evaluate the prognostic value of the ATP5B score, we used the Kaplan–Meier method to estimate the probability of metastasis-free survival. To confirm these findings, the Kaplan–Meier curves were compared using the log-rank (Mantel–Cox) test. P-values were calculated according to the standard normal asymptotic distribution, using a cutoff of 0.05 for significance. Survival analysis was performed in GraphPad Prism.

## Results

### Identification of an aptamer recognizing aggressive cancer

**Fig. 1:**
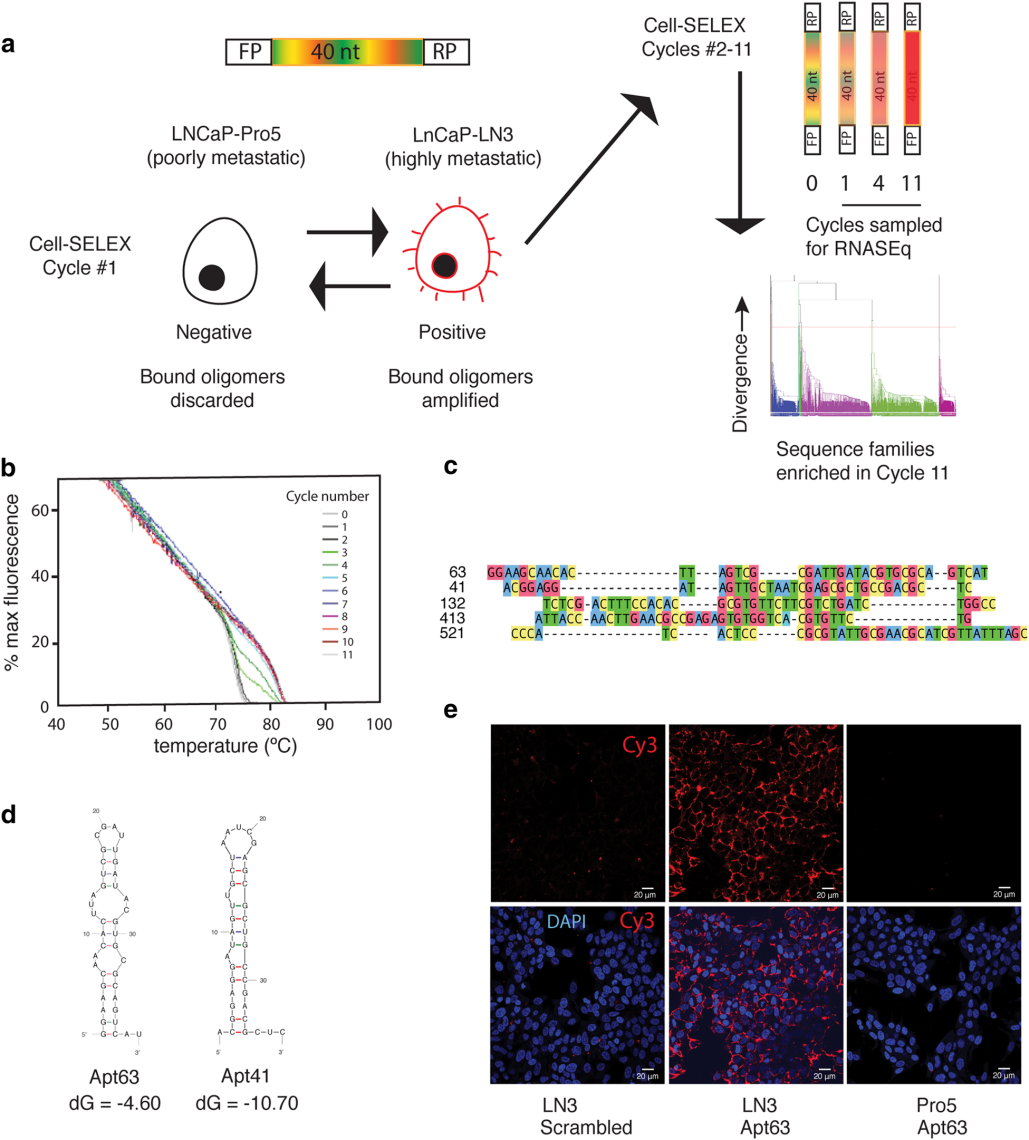
Identification of Aptamer 63 (Apt63) by differential Cell-SELEX. **a** Schematic of the Cell-SELEX screening process (top). Representation of the RNA molecules library featuring a central 40 random nucleotides (multicolor), flanked by forward (FP) and reverse (RP) primer sequences. The library was screened for differential binding to surface feature(s) unique to LNCaP-LN3 prostate cancer cells. Negative selection was performed using poorly metastatic LNCaP-Pro5 cells (smooth black) and positive selection was performed on the highly metastasis-prone LN3 subclone (spiky red). Sequential negative and positive selection cycles enrich the aptamer pool for LN-binding sequences (top right). RNA aptamer pools were sampled after cycles 1, 4, and 11 and sequenced. Sequences enriched at cycle 11 relative to earlier cycles were aligned and clustered (bottom right). **b** RoT curve analyses for cycles 0–11. Progressive increase in binding affinity is seen as the complexity of the aptamer pool decreases. Enrichment for high-affinity binding is detectable as early as cycle 3. **c** Alignment of 5 sequences from **(a)** that was tested for selective in vitro binding to LN3 cells. **d** The secondary structure of 2 aptamer sequences showing selective binding to LN3 vs. Pro5 cells was determined using mFold Web Server (The RNA Institute, University at Albany, State University of New York). **e** Representative confocal images of LN3 and Pro5 cells bound by Cy3-labeled Apt63 or AptScr. (Color figure online)

As an initial approach to discovering features of cancers with high metastatic risk, we performed differential Cell-SELEX comparing two subclones of a single prostate cancer line (LNCaP) with divergent metastatic potential. Parental LNCaP and Pro5 variant lines, which are poorly metastatic, were used for library subtraction, while the aggressive LN3 line was used for positive screening. 11 cycles of negative and positive selection were performed (Fig. 1a). Ongoing enrichment of high-affinity LN3-specific aptamers was monitored by SYBR® Green fluorescence as an indicator of annealing. Figure 1b shows RoT curve analyses at each cycle, demonstrating a progressive increase in binding affinity and decreasing the complexity of aptamers as selection progress. Aptamer pools were sampled at cycles 0, 1, 4, and 11 and sequenced. Sequences showing a frequency higher than 1/10^6^ in the last analyzed cycle (i.e., cycle 11) were selected for further investigation. After this filtering step, 691 unique sequences were clustered into families using Clustal Omega software [34] (Fig. 1c), and representative aptamers from each of 5 selected families were selected for further testing.

The selected RNA aptamers, together with scrambled controls, were labeled with Cy3 and incubated with live LN3 and Pro5 cells. Aptamers #63 and #41 showed strong binding to LN3, while only background fluorescence was seen with the other aptamers and control sequences (Fig. 1d and Online Resource 4). Pro5 cells were not bound by either aptamer or by scrambled controls (Fig. 1d and Online Resource 4). Because of the greater intensity of Apt63 fluorescence, this sequence was chosen for further testing.

**Fig. 2:**
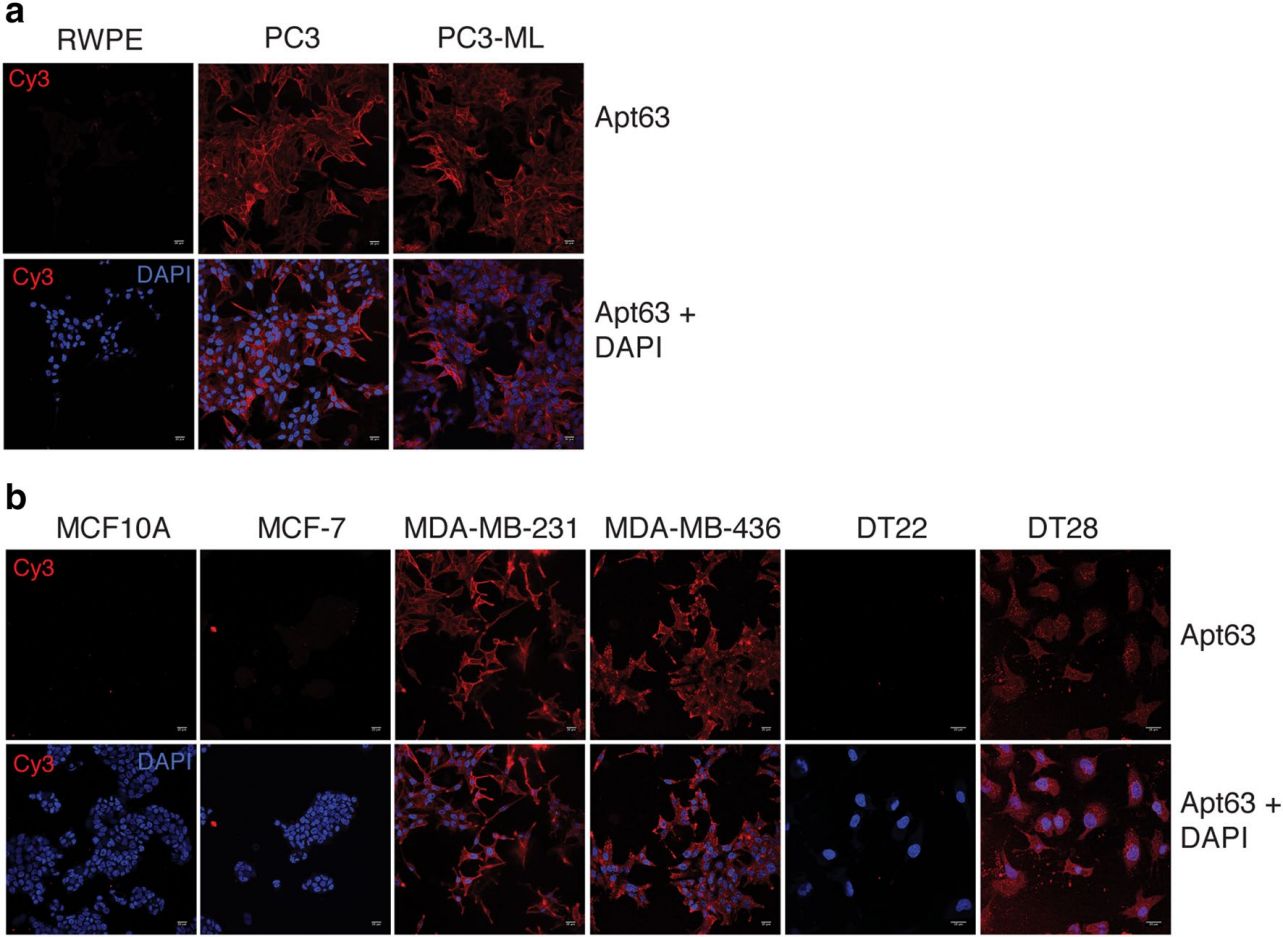
Discriminative staining of aggressive breast cancer by Aptamer 63. The indicated cancer cell lines in live in culture were incubated with 1 nmol of Cy3-labeled Apt63 (top) or a AptScr (below). **a** Aggressive PC-3 and PC-ML show positive staining by Apt63; prostate epithelial cell line RWPE does not show positive staining. **b** Weakly metastatic MCF-7, aggressive MDA-MB-231 and MDA-MB-436 breast cancer cell lines, and aggressive DT28 dissociated tumor line show membrane-specific staining; a non-tumor-forming breast epithelial cell line MCF10A and non-metastatic DT22 tumor line do not have positive staining. Original confocal imaging magnification: × 20. (Color figure online)

We next asked whether Apt63 would preferentially interact with other highly metastatic cell lines derived from other tissues, including human prostate, human breast, and murine breast cancers. Non-tumorigenic and poorly metastatic cell lines were used for comparison (Fig. 2). As with LN3, the aggressive prostate cancer cell lines PC-3 and PC3-ML were strongly labeled by Apt63, while the non-tumorigenic prostate epithelial cell line RWPE-1 was not (Fig. 2a). The readily metastasizing MDA-MB-231 and MDA-MB-436 breast cancer cell lines were also strongly labeled by Apt63, but the non-tumorigenic breast epithelial cell line MCF10A was not labeled, and the poorly metastasizing MCF-7 line was weakly bound by Apt63. The primary dissociated breast tumor line DT28, which metastasizes efficiently, was strongly labeled by Apt63, but the non-metastasizing DT22 line was not [35]. Apt63 also efficiently discriminated between murine breast cancer cell lines with different metastatic potentials (Online Resource 5), indicating inter-species conservation of the binding target. Live cells stained with Apt63 showed a punctate pattern that appeared to be concentrated at the plasma membrane, with some variation in labeling intensity (Fig. 2a, b). These findings suggest that the target recognized by Apt63 is located on the cell surface and is a common feature among cell lines with high metastatic potential.

### Apt63 binding is selectively cytotoxic to cancer cells in culture and in vivo

**Fig. 3:**
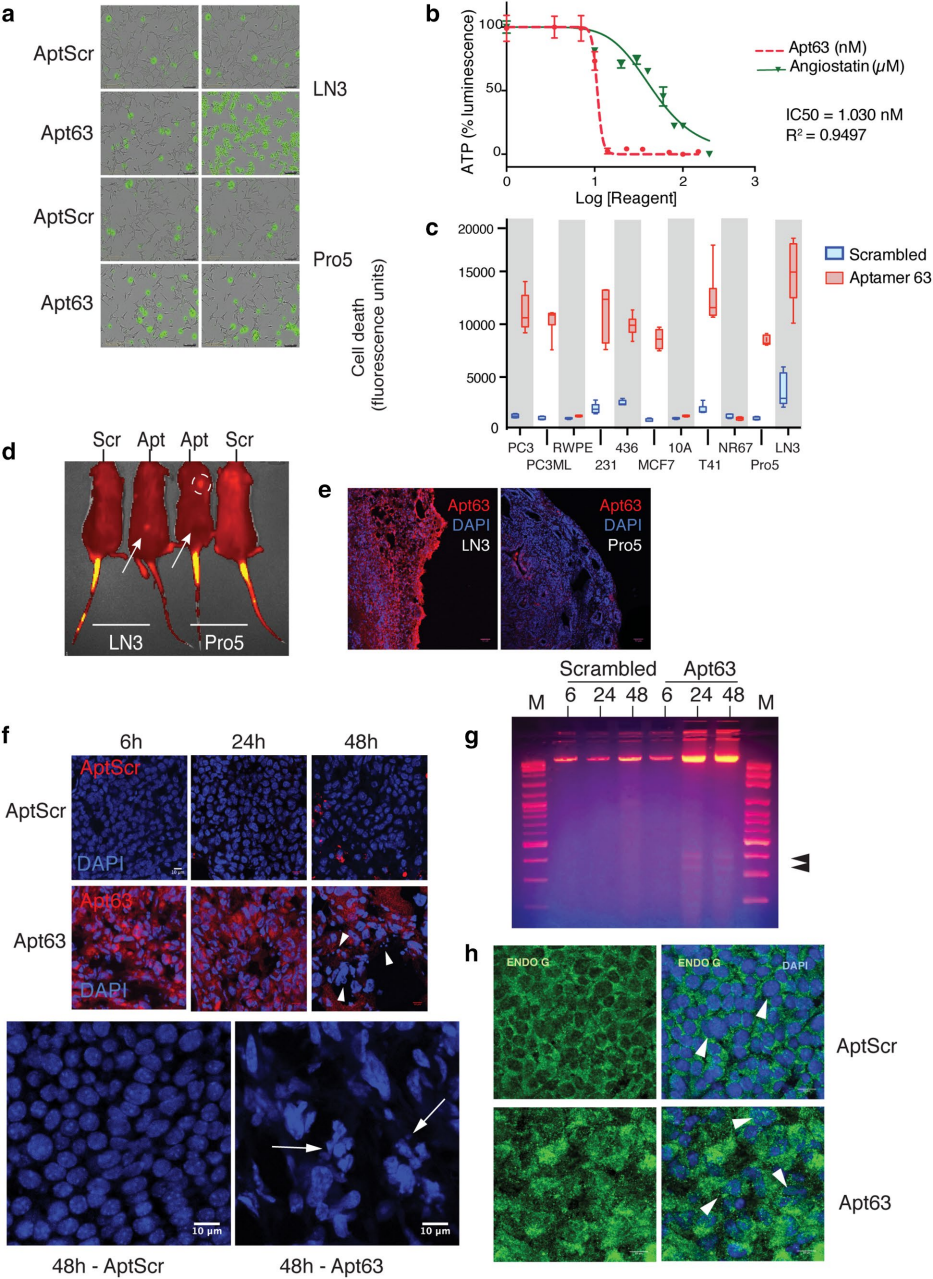
Apt63 binding is selectively cytotoxic to cancer cells in culture and in vivo. **a** Rapid in vitro cytotoxicity induced by Apt63. Cell death was monitored in real time by SYTOX® Green fluorescence as described in “Materials and methods.” Representative photographs after 10 min (left column), and after 2-h post-treatment (right column). Original magnification: × 20. **b** Concentration dependence of Apt63 cytotoxicity. Apt63 (unlabeled) or angiostatin was added to the cells at the indicated concentrations and luciferin luminescence was measured at 2 h in an EnVision™ plate reader. Readings were normalized to untreated cells and plotted using GraphPad Prism 8 Software. Apt63 IC50 = 1.030 nM. IC50 for angiostatin = 1.66 micromolar (> 10^3^ × higher). **c** Cell selectivity of Apt63 cytotoxicity. The indicated cell lines were incubated with Apt63 or a scrambled aptamer (AptScr) in the presence of SYTOX® Green. Y-axis indicates absolute fluorescence units at 3 h. **d, e** Selective Apt63 uptake by LN3 xenograft tumors after intravenous injection in vivo. LN3 and Pro5 orthotopic tumor xenografts were established in mice as described in “Materials and methods,” and Alexa Fluor 647-labeled Apt63 or AptScr was injected via tail vein. **d** Representative in vivo images, 3.5 h post injection on an IVIS Spectrum in vivo imaging system (Perkin Elmer). Arrows indicate the position of xenograft tumors in Apt63-injected mice; circle indicates site of a lung abscess identified postmortem. **e**. Selective uptake of Apt63 by LN3 cells. Frozen sections of tumors removed from animals 4 h after delivery of labeled aptamer were counterstained with DAPI. Representative fluorescence images are shown. **f** Apt63 binds to and induces nuclear fragmentation in MDA-MB-231 breast xenograft tumors in vivo. Orthotopic MDA-MB-231 xenografts were established as described in “Materials and methods.” Mice received a single injection of 1 nmol Apt63 by tail vein as in (**d**), above, and were euthanized 6, 24, 48 h later. Xenograft frozen sections were prepared as in (**e**). Representative images AptScr- (top) and Apt63- (center) labeled tumors at each time point. Note retention of fluorescent label and increased nuclear fragmentation in Apt63-exposed xenografts (arrowheads). (Bottom) Enlarged images showing DAPI-stained xenograft tumors at 48 h after injection of AptScr and Apt63. Arrows point to fragmented MDA-MB-231 nuclei in Apt63-exposed tumors. **g** Nucleosomal cleavage pattern in Apt63-treated cells. DNA fragmentation in Apt63-treated tumors was confirmed by electrophoresis of total genomic DNA. Electrophoresis of tumor DNA shows accumulation of 300–400 bp DNA cleavage products (arrowheads). **h** Apt63 induces EndoG translocation from mitochondria to nuclei. Xenograft frozen sections at 24 h were stained with rabbit polyclonal EndoG antibody (ab9647, ABCAM) and counterstained with DAPI. (Color figure online)

We speculated that the target of Apt63 on LN3 cells might be functionally important in promoting its metastatic phenotype, possibly as a survival factor, and that binding by the aptamer might impair this function. Accordingly, we tested for direct Apt63 cytotoxicity in vitro, using two methods. First, real-time cytotoxicity was monitored in Apt63-exposed LN3 cells by SYTOX® Green uptake and fluorescence, using an IncuCyte® S3 Live-Cell Analysis System. The SYTOX® Green nucleic acid dye is excluded by healthy cells with normal membrane permeability but diffuses passively through damaged membranes. After addition of Apt63 or a scrambled aptamer (AptScr), together with SYTOX® Green, fluorescent images were recorded every 5 min for the duration of the experiment (Fig. 3a; see also the time-lapse video in Online Resource 6). No difference in cell death was seen among the various conditions at 20 min (Fig. 3a). By 100 min, most LN3 cells exposed to Apt63 were brightly fluorescent, indicating cytotoxicity, but there was no change in ongoing basal death rates of Pro5 or AptScr-treated LN3 cells (Fig. 3a). These findings suggest that Apt63 induces rapid cell death upon engagement of an LN3-enriched epitope, leading to membrane compromise and cell death within 2 h. Next, we estimated the dose dependence of Apt63 cytotoxicity in LN3 cells using ATP content as an indicator of cell viability. Cells were exposed to a range of concentrations of Apt63 and media ATP fluorescence determined using a CellTiter-Glo® system as described in “Materials and methods.” A relatively sharp decrease in cell viability is observed at an approximate IC50 = 1.030 nM (*R*^2^ = 0.9497, Fig. 3b). For comparison, the IC50 for angiostatin in this assay was 1.66 µM (*R*^2^ = 0.884. Fig. 3b).

To determine whether Apt63 binding and cytotoxicity were correlated in other cell lines, we grew human PC3, PC3ML, RWPE, MDA-MB-231, MDA-MB-436, MCF7, MCF10A, and mouse T41 and NR 67 cell lines, along with the original LN3 and Pro5 cell lines, in 96-well plates for 24 h. Each cell line was treated with unlabeled 1 nM of Apt63 and 5 nM SYTOX® Green dye for 3 h. Binding of Apt63 to live cells in culture correlated closely with rapid cytotoxicity as shown by SYTOX® Green fluorescence (Fig. 3c, red bars; compare Fig. 2a). No cell death was seen with AptScr (blue bars). Non-tumorigenic epithelial cell lines RWPE and MCF10A, which do not bind Apt63, were completely resistant, while cell lines with weak staining had correspondingly reduced susceptibility (e.g., MCF-7) (Fig. 3c). The residual toxicity of Apt63 in these weakly staining cells could reflect the presence of small subpopulations of vulnerable cells. Overall, however, these findings suggest that Apt63 cytotoxicity is sequence-specific and dependent on the presence of a specific epitope found on multiple cancer cell types.

We then asked whether Apt63 could exert sequence-specific binding and toxicity for cells grown as xenograft tumors in vivo. In initial experiments, we tested whether LN3 and Pro5 tumor cells would differentially take up Apt63 after intravenous injection. Mice bearing LN3 and Pro5 xenograft tumors were injected with a single dose of 1 nmol of Alexa Fluor™ 647-labeled Apt63 or AptScr in 200 µL of PBS into the tail vein. Between 10 min and 3.5 h post injection, Apt63 uptake could be detected in LN3 but not Pro5 xenografts in vivo (Fig. 3d) and in frozen sections of the same tumors (Fig. 3e). No Apt63 uptake was observed in Pro5 xenografts (Fig. 3d), suggesting that Apt63 selectively binds to and accumulates in tumors expressing its plasma membrane target. We next injected Alexa Fluor^TM^-labeled Apt63 or AptScr into mice bearing MDA-MB-231 xenograft tumors in the mammary fat pad. Mice were monitored and euthanized at 6, 24, and 48 h after a single tail vein injection of Apt63 or AptScr, and frozen sections of tumors were imaged as described above. The fluorescent label was retained by Apt63-exposed MDA-MB-231 xenografts for up to 48 h post injection, while only background AptScr signal was detectable at any point (Fig. 3f).

In the same images, we noted an increase in nuclear fragmentation in MDA-MB-231 xenografts by 24 h after injection of Apt63, relative to AptScr (Fig. 3f). We further explored this by electrophoresis of tumor DNA (Fig. 3g), which revealed a distinct nucleosomal DNA cleavage pattern in Apt63-treated tumors. The cleavage pattern resembled that produced by endonuclease G (endoG), a nuclease that is released from the inter-mitochondrial membrane space during oxidative stress and translocates to the nucleus to initiate a caspase-independent apoptotic pathway [36, 37]. Consistent with this, Apt63-treated MDA-MB-231 tumors showed considerable nuclear endoG staining by 24 h, while AptScr-treated tumors did not (Fig. 3h, arrowheads). These effects were not accompanied by any visible cytotoxicity toward adjacent non-tumor tissues, or any obvious adverse effects on the mouse overall conditions during the 48 h after injection. We interpret these results to show that Apt63 binds preferentially to breast and prostate tumor cells that express its plasma membrane target, and that Apt63 induces cell death upon binding, through a mechanism that involves release and nuclear translocation of endoG.

### The target of Apt63 is the beta subunit of F_1_F_o_ ATP synthase (ATP5B)

**Fig. 4:**
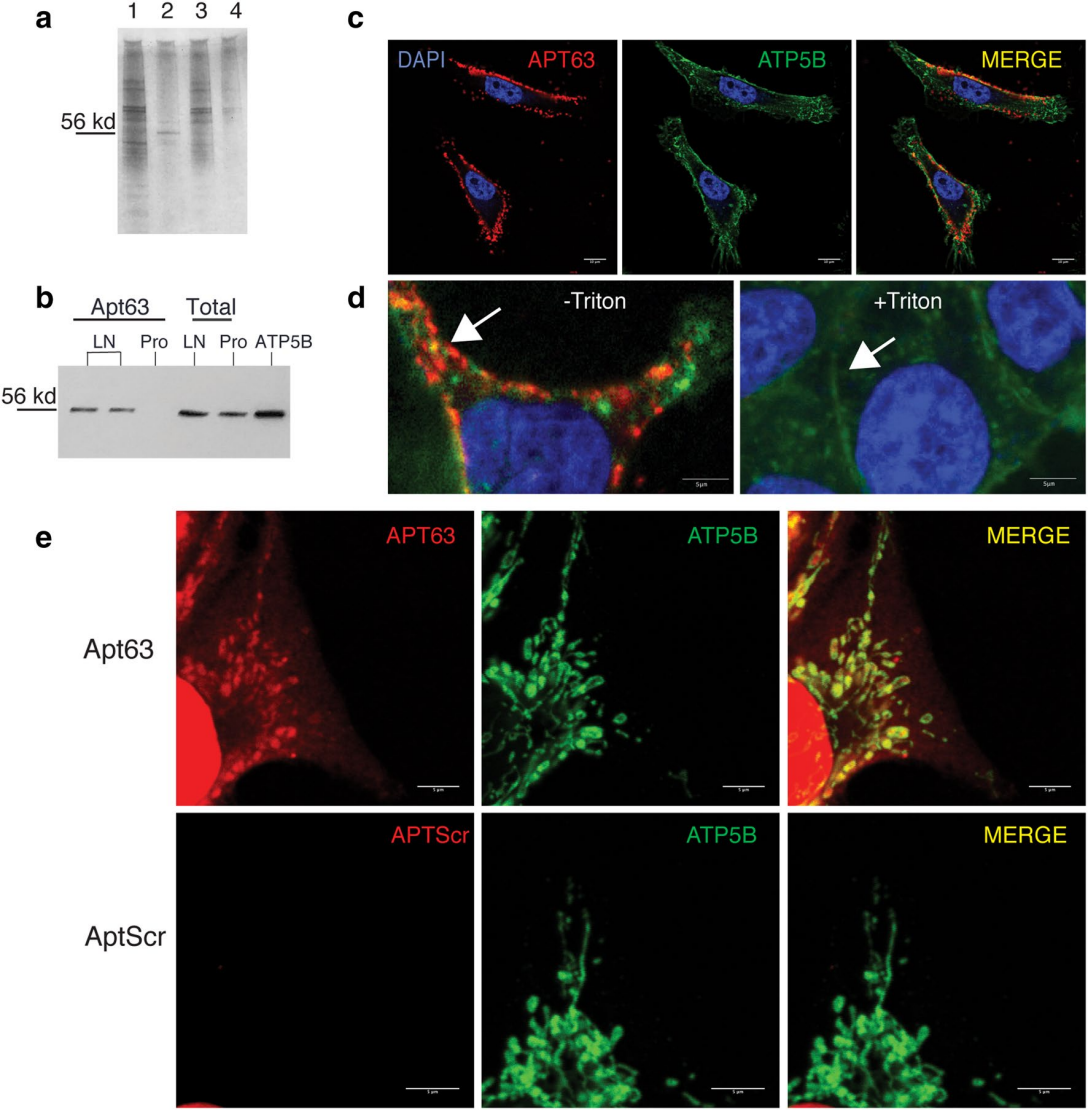
Identification of Apt63 target as cell surface F_o_F_1_-ATP synthase β-subunit ATP5B. **a** Apt63 associates with a 56kd protein in the LNCaP-LN3 plasma membrane. Representative SDS-PAGE gel of proteins immunoprecipitating with Apt63 from LN3 and Pro5 cell lines. Lane 1: LN3 whole cell lysate; Lane 2: LN3, membrane-enriched fraction; Lane 3: Pro5 total cell lysate; Lane 4: Pro5 membrane-enriched fraction. Equal amounts of protein were loaded in each lane. Bar at 56 kd indicates the predominant LN3 aptamer-associated band subjected to mass spectroscopy as described in “Materials and methods” and Table 1. **b** Confirmation of F_o_F_1_-ATP synthase β-subunit as aptamer target. Membrane fractions of LN3 and Pro5 prostate cells were separated as in **(a)**. Plasma membrane-bound Apt63/target complexes (lanes 1–3), and whole cell lysates (lanes 4–5) were obtained from indicated cell lines and immunoblotted with an anti-ATP5B monoclonal antibody. A single ~ 56 kd protein band co-migrating with recombinant ATP5B (lane 6) is present on the membranes of LN3 (lanes 1, 2) but not Pro5 cells (lane 3). Similar bands are present in whole cell lysates from both cell lines, reflecting the mitochondrial protein (lanes 4–5). **c** Apt63 co-localizes on plasma membrane with anti-ATP5B antibody. ATP5B antibody (green) and Apt63 (red) were bound to live LN3 cells, followed by fixation and imaging by confocal microscope. **d** The Apt63 target is extractable by detergent treatment of fixed cells. (left) Cy3-Apt63 (red) and plasma membrane marker WGA (green) were incubated with live cells followed by fixation and imaging as in **(c)**. (right) Similarly treated cells, except that fixed slides were subjected to a short 0.05% Triton X-100 treatment. Note loss of Cy3 Apt63 signal from plasma membrane. **e** Apt63 stains mitochondria in permeabilized cells. Cells were fixed, then permeabilized with 0.05% Triton X-100 and incubated with Cy3-Apt63 or Cy3-AptScr and ATP5B antibody. In these permeabilized LN3 cells, Apt63 and ATP5B antibody staining are co-localized within mitochondria. (Color figure online)

To obtain an enriched fraction of the aptamer target on LN3 plasma membranes, we used a protocol combining a short detergent treatment with mild hypotonic lysis to segregate aptamer-bound membrane proteins from other cell components (see “Materials and methods”). Electrophoresis of this fraction yielded a single predominant protein band (Fig. 4a, lane 2.) A similar protein band was identified in PC-3 membrane fractions (not shown). These bands were isolated and sent for protein sequencing by mass spectrometry (*n* = 2 samples from LN3, *n* = 1 from PC3). The top hit in all 3 samples was ATP5B, with 28.36% of the ATP5B protein sequence detected (Table 1).

**Table 1:** Mass spectroscopy of membrane proteins immunoprecipitated from LN3 and PC-3 by Apt63. Separate experiments and MS runs were performed for each cell line. The amino acid sequence of ATP5B is shown below with mapped peptides in italics

Cell line	Protein mass	Amino acids	Amino acids identified	Protein coverage (%)	Peptides identified
LN3	56524.61	529	118 AA	22.31	14
PC-3	56524.61	529	109 AA	20.60	13
		Total	150 AA	28.36	27

MLGFVGRVAA APASGALRRL TPSASLPPAQ LLLRAAPTAV HPVRDYAAQT SPSPKAGAAT GRIVAVIGAV VDVQFDEGLP PILNALEVQG RETRLVLEVA QHLGESTVRT *IAMDGTEGLV R*GQK*VLDSGA PIKIPVGPET LGRIMNVIGE PIDERGPIK*T KQFAPIHAEA PEFMEMSVEQ EILVTGIK*VV DLLAPYAK*GG K*IGLFGGAGV GKTVLIMELI NNVAK*AHGGY SVFAGVGERT REGNDLYHEM IESGVINLKD ATSK*VALVYG QMNEPPGAR*A RVALTGLTVA EYFRDQEGQD VLLFIDNIFR *FTQAGSEVSA LLGRIPSAVG YQPTLATDMG TMQER*ITTTK KGSITSVQAI YVPADDLTDP APATTFAHLD ATTVLSR*AIA ELGIYPAVDP LDSTSRI*MDP NIVGSEHYDV ARGVQKILQD YKSLQDIIAI LGMDELSEED KLTVSRARKI QRFLSQPFQV AEVFTGHMGK LVPLKETIKG FQQILAGEYD HLPEQAFYMV GPIEEAVAKA DKLAEEHSS

To confirm the identity of the aptamer target, we performed a western blot analysis of aptamer-associated cell membrane proteins and total cell lysates using an ATP5B antibody (Fig. 4b). A single protein band was present in Apt63-bound membrane fractions of LN3 (Fig. 4b, lanes 1, 2) but not Pro5 cells (Fig. 4b, lane 3). The same band was readily detected in whole cell lysates of both cell lines (Fig. 4c, lanes 4, 5), and co-migrated with recombinant ATP5B protein (4c, lane 6). ATP5B antibody and Apt63 co-localized on the surface of intact LN3 cells (Fig. 4d), and within mitochondria in permeabilized LN3 cells (Fig. 4e), consistent with ectopically expressed ATP5B on the plasma membrane as a common target of the antibody and Apt63. This surface target could be extracted by detergent treatment (Fig. 4d, right), further supporting the plasma membrane location of ATP5B.

### Membrane ATP5B as a correlate of tumor metastasis in clinical populations

**Fig. 5:**
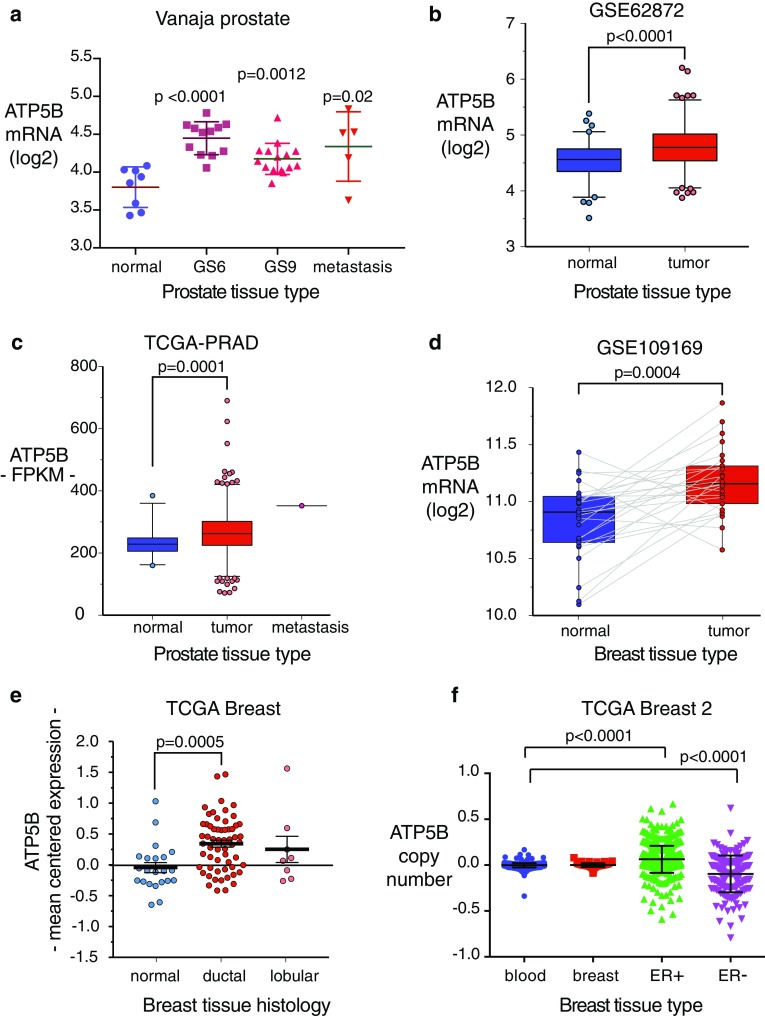
ATP5B expression is increased in prostate and breast cancer vs. normal tissue. Datasets were reviewed as described in “Materials and methods.” **a** ATP5B expression is increased in prostate adenocarcinoma compared with normal prostate tissue (*p* = 3.54e−4). GS = Gleason stage. Data from Vanaja et al. [74]. **b** ATP5B overexpression in GSE62872 prostate samples compared with normal prostate tissues (*n* = 424; *p*-value < 0.0001). **c** ATP5B overexpression in TCGA-PRAD samples compared with normal prostate tissues (*n* = 551; *p*-value = 0.0001). **d** ATP5B overexpression in GSE109169 breast cancers compared with their paired normal breast counterparts (*n* = 50; *p*-value = 0.0004). Gray lines link normal and tumor paired samples. **e** ATP5B overexpression in breast ductal cancers as compared to normal mammary tissues (*n* = 544; *p*-value = 0.0005). Data obtained from TCGA-BRCA database. **f** ATP5B copy number variation in breast cancer compared to normal blood cells. Copy number is significantly increased in ER + tumors. Data from TCGA Breast 2 dataset. (Color figure online)

**Fig. 6:**
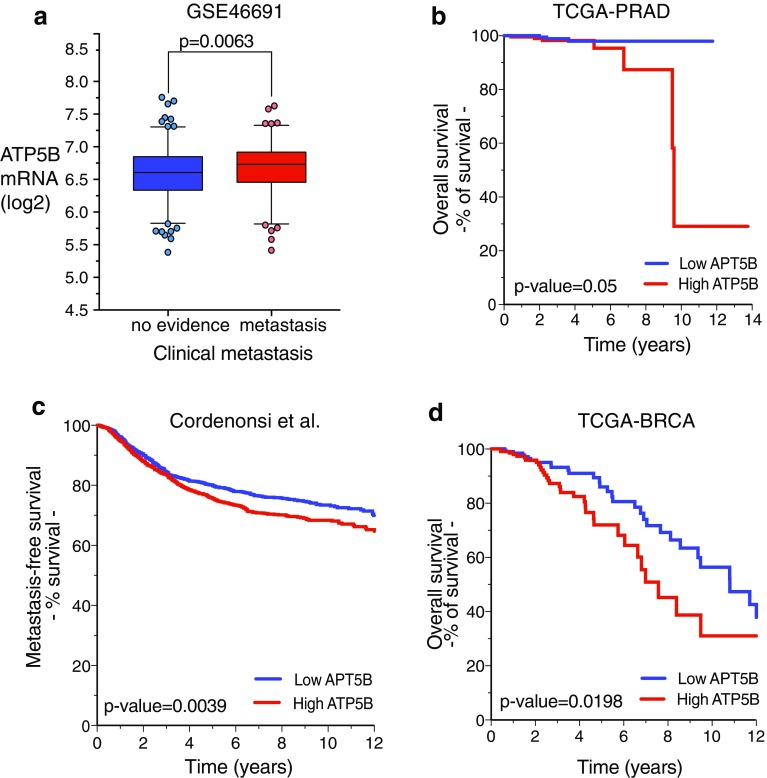
Increased ATP5B expression is associated with greater risk of metastasis and reduced overall survival in prostate and breast cancer. **a** ATP5B expression is increased in primary prostate tumors that later metastasize. Primary prostate cancers with metastatic disease progression were compared to samples with no evidence of metastasis. Data were obtained from GSE46691 [27] (*n* = 545; *p*-value = 0.0063). **b** High expression of ATP5B mRNA correlates with decreased overall survival in prostate cancer patients of the TCGA-PRAD dataset (*n* = 551). Patients with high and low ATP5B expression are shown in red and blue, respectively (see “Materials and methods”). Patients with high expression (*n* = 250) had lower overall survival probability over time (log rank *p*-value = 0.050). **c** High expression of ATP5B mRNA correlates with reduced metastasis-free survival in patients of a compendium of 3661 breast cancer samples. Patients with high and low ATP5B expression are shown in red and blue, respectively (see “Materials and methods”). Patients with high expression (*n* = 1656) had higher probability to develop metastasis over time (log rank *p*-value = 0.0039). **d** High expression of ATP5B mRNA correlates with poor overall survival in breast cancer patients of the TCGA-BRCA dataset (*n* = 522). Patients with high and low ATP5B expression are shown in red and blue, respectively (see “Materials and methods”). Patients with high expression (*n* = 263) had lower overall survival probability over time (log rank *p*-value = 0.0198). (Color figure online)

It is not clear how ATP5B gene expression and ecto-ATP5B levels are related in any given cell type; ATP5B protein is subject to substantial post-translational and functional regulation, including plasma membrane redistribution [16, 38–41]. However, several components of the ATP synthase complex have been reported to be upregulated in cancer [40, 42, 43]. We therefore asked whether ATP5B expression was associated with cancer phenotypes in clinical populations by comparing ATP5B transcript levels in tumor vs. normal tissue in multiple prostate and breast cancer datasets. ATP5B expression was significantly higher in primary tumors when compared with normal tissues in both prostate (Fig. 5a–c) and invasive ductal breast cancer (Fig. 5d, e). In tandem with this, mean ATP copy number was significantly increased in ER-positive tumors, and in a subset of ER− (Fig. 5f), although interestingly the average copy number for ER− tumors was reduced. For both types of cancer, above-median ATP5B expression was associated with significantly decreased metastasis-free (Fig. 6a, b) and overall (Fig. 5c, d) survival. These findings are consistent with a role for ATP5B, along with other members of the complex, in supporting metastatic progression.

**Fig. 7:**
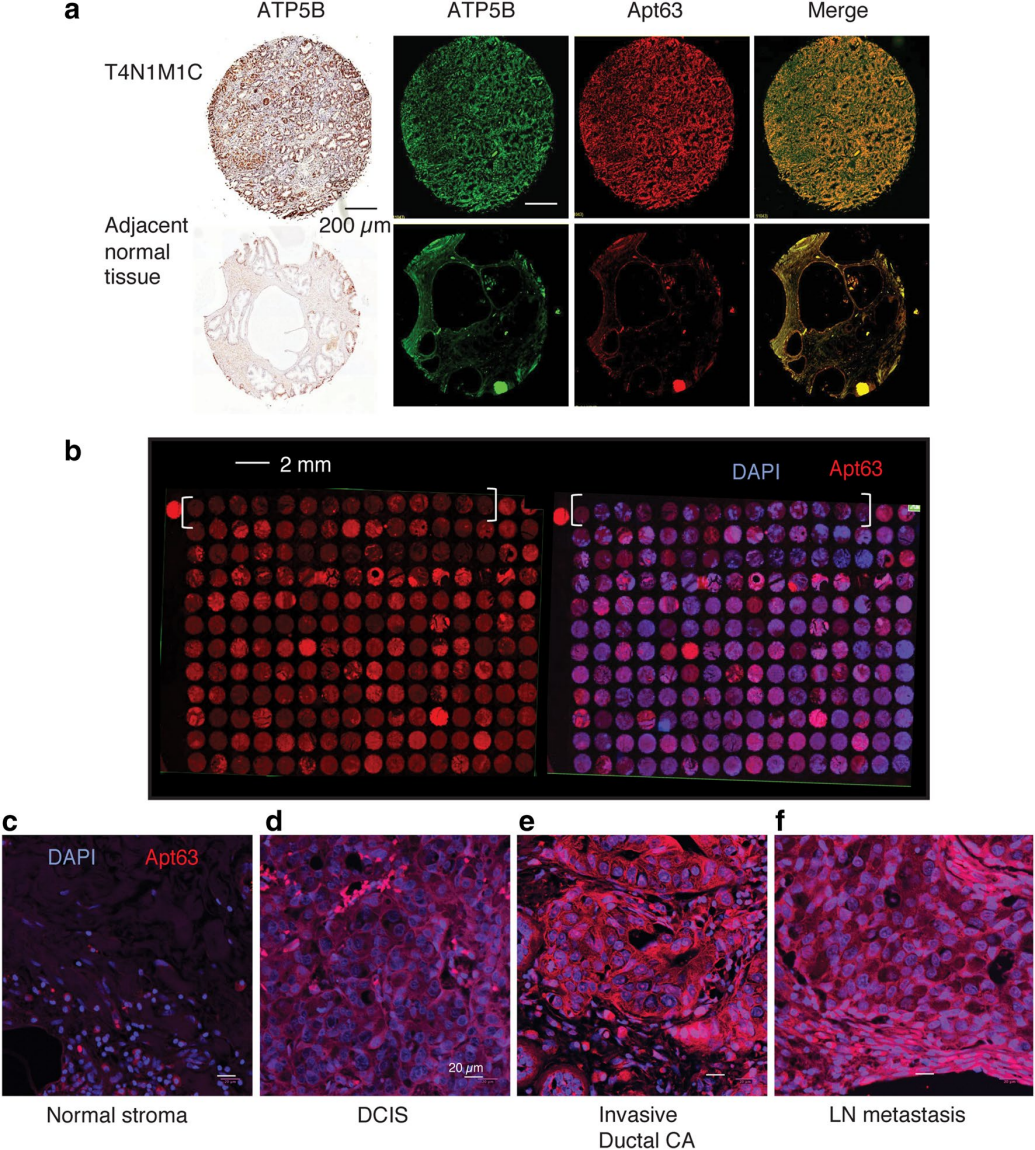
Apt63 binds to a subset of aggressive breast tumors and co-localizes with ATP5B. **a** Primary tumor core biopsies (top row) and adjacent normal tissue (bottom row) with anti-ATP5B (Abcam #14730) using HRP (far left) or Alexa Fluor® 647 ATP5B antibodies (ab223436, ABCAM) (near left). ATP5B (assigned green color) and Cy3-Apt63 (red color) extensively co-localize both at cell membranes and in the cytosol. **b** Heterogeneous binding of Cy3-Apt63 to primary breast tumors and lymph node metastases. Brackets indicate normal adjacent tissues in the top row. **c–f** Representative high-resolution confocal images of normal tissue **(c)**, ductal carcinoma in situ (DCIS) **(d)**, invasive ductal carcinoma (CA) **(e)**, and lymph node metastases (LN) **(f)**, imaged with Cy3-Apt63. A membrane staining pattern, as well as cytosolic staining, can be seen in **(e)** and **(f)**. Original magnification: x20. Results shown are representative of findings in a total of 417 breast biopsies. (Color figure online)

To characterize ATP5B protein content in human breast and prostate cancer samples, we used Apt63 to label prostate and breast cancer tissue microarrays (TMAs) representing a range of tumor grades and stages. As in the xenograft studies shown above, we confirmed that Apt63 staining co-localized with staining by a monoclonal ATP5B antibody within tumor tissue, while normal adjacent stroma was only minimally bound by either reagent (Fig. 7a). Both cytosolic and membrane staining patterns could be identified by high-resolution confocal microscopy. We observed considerable sample-to-sample heterogeneity of staining patterns across different categories of breast cancer; in some tumors, Apt63 predominantly labeled cytosolic components, including mitochondria, while in others, a clear plasma membrane pattern was identified (Fig. 7b). Staining of normal tumor-adjacent tissue was consistently weak (Fig. 7b, brackets). Using a semi-quantitative score for the presence or absence of membrane-bound ATP5B (see Online Resource 2), we saw no consistent association between the presence of ecto-ATP5B and tumor grade, PAM50 subtype, or hormone receptor status. However, plasma membrane staining of breast cancer cells by Apt63 was strongly and positively associated with tumor stage (*r* = 0.997, *p* = 3.12E−03), appearing in 42/46 of lymph node metastases, 0/12 normal breast tissue samples, and intermediate values in DCIS and invasive carcinomas (summary is presented in Table 2 and in Online Resource 3). These results provide further support for a functional relationship between plasma membrane ATP5B, as indicated by binding by Apt63, and breast cancer metastasis.

**Table 2:** Tissue biopsies by pathology groups and scoring for Apt63 membrane staining

Tissue	Apt63 positive	Total samples	Percent (%)
Normal breast tissue	0	12	0.00
Benign tumors	4	31	12.90
Cancer-adjacent normal breast tissue	3	19	15.79
Breast Invasive lobular CA*	11	30	36.67
Ductal CA* in situ	10	22	45.45
Breast invasive ductal CA*	109	198	55.05
Lymph node metastases	42	46	91.30

High-resolution confocal images of Cy3-Apt63 TMAs were examined for membrane-pattern staining as illustrated in Fig. 7. Apt63-specific membrane staining correlates with cancer stage, *r* = 0.997 and p-value 3.12E-03(*carcinoma). Group of benign tumors represented by adenosis, hyperplasia, and fibroadenosis. Online Resource 3 contains the complete list of 416 core biopsies with stage, grade, pathology diagnosis, and Apt63 score

## Discussion

Here we show that ectopic plasma membrane ATP5B, a subunit of F_1_F_o_-ATP synthase, denotes a high metastasis-risk phenotype in breast and prostate cancer, and a vulnerability of cancer cells *in vivo*. F_1_F_o_ ATP synthase is a highly conserved enzyme complex residing on the inner mitochondrial membrane, where it conducts the final step in oxidative ATP production. Its 30 protein components are organized into two domains, the F_o_ proton-translocating domain and the F_1_ catalytic domain [44, 45]. Three pairs of ATP5A and ATP5B subunits form the catalytic core of F_1_ in the inner mitochondrial membrane, generating ATP molecules as H + transits the F_o_ pore. Defects in ATP synthase contribute to diseases including microbial infection, immune deficiency, neuropathies, obesity, diabetes, and cancer [35, 46, 47].

A plasma membrane-located ATP synthase (ecto-ATP synthase) was initially discovered as a cancer neoantigen more than 20 years ago [48]. Fully functional ATP synthase complexes have been identified on the plasma membrane of certain normal and many tumor cells, and may either hydrolyze or synthesize ATP [10, 11]. Ecto-ATP synthase has been proposed to act as a receptor for apo-A1 and thereby to regulate HDL uptake by hepatocytes [49, 50] and to promote endothelial progenitor cell proliferation and angiogenesis [51]. Angiostatin has been shown to bind to ecto-ATP synthase and disrupt its ATP synthetic activity, contributing to its anti-angiogenic effects [10, 52]. However, angiostatin is able to exert these functions through other receptors on the cell surface, including c-met [53], proteoglycan NG2 [54], and annexin II [55]. The importance of these functions of ecto-ATP synthase in normal cells remains to be fully elucidated [38, 56].

ATP5B emerged in our unbiased screen as a plasma membrane feature that distinguishes the aggressive LNCaP-LN3 cell line from isogenic LNCaP and LNCaP-Pro5 cells, which metastasize infrequently [19]. Collectively, our data suggest that acquiring this feature may have enabled the metastatic phenotype of the LN3 subclone. Despite substantial effort, no other specific drivers of the aggressive LN3 phenotype have been identified. LN3 cells grow well in the absence of androgen, but do not have androgen receptor amplification [57]; LN3 also exhibits higher resistance to apoptosis, associated with up-regulation of anti-apoptotic BCL-2 and down-regulation of BAK and BAX [57]. LN3s express higher levels of macrophage-inhibitory cytokine-1 (MIC1/GDF15) [58], the chaperone gp96 [59] and VEGFA [60], and have greater tumor vascularity [59, 60] than non-metastatic LNCaP lines. No genetic differences have been shown to explain these properties, although LN3 displays unique deletions in 16q23–qter and 21q of unknown functional significance [61] and lacks a missense mutation in PlexinB1 found in parental LNCaP cells that appears to be silent [62]. Previous proteomic analyses found no features distinguishing LN3 from the less-aggressive isogenic lines [63]. The same study found that endoplasmic reticulum protein ERp5 is overexpressed and displayed on the plasma membrane of both LN3 and Pro5 cells, demonstrating that cycling of intracellular peptides to the plasma membrane is not a rare event during tumorigenesis [63].

Considerable evidence links ecto-ATP synthase to aggressive cancer cell growth. Plasma membrane-associated ATP5 subunits, including ATP5B, have been correlated with more-aggressive, larger and more advanced tumors, in multiple cancers including breast, lung, and prostate [17, 42, 64]. In our breast cancer TMA analysis of Apt63 binding, including biopsies representing 416 subjects, surface ATP5B appears to define a unique subset of highly aggressive breast and prostate cancers, present on 45% of DCIS and 55% of invasive ductal carcinomas, and on almost all (91.3%) lymph node metastases. Apt63 staining did not appear to align with tumor size or hormone receptor status, suggesting that ecto-ATP5B denotes an independent tumor phenotype. Surface ATP5B also appears to be important as a tumor-specific survival factor: cancer cells expressing ecto-ATP5B were rapidly killed by Apt63 binding, undergoing nuclear translocation of endonuclease G and DNA fragmentation, while adjacent normal tissues were spared. This selective toxicity could mean that certain breast and prostate tumors are dependent on the presence of functional ecto-ATP synthase, and points to a vulnerability not shared by non-transformed cells.

The biological importance of ecto-ATPase has been explored using a range of physiological and synthetic ligands, including angiostatin, plasminogen, monoclonal antibodies, peptides, and small molecules binding to the F_1_ module [12, 14, 16, 64–67]. The effects of these agents are both cell type and ligand-specific, but most reduce extracellular ATP production and cell proliferation, and some initiate programmed death. In HUVECs, which express high levels of ecto-ATP synthase, angiostatin inhibited cell proliferation and ATP production, but was not cytotoxic [10]; in A549 lung cancer cells, both angiostatin and a polyclonal anti-ATP5B antibody blocked ATP synthesis, induced intracellular acidification, and triggered cell death [68]. A monoclonal ATP5B antibody (McAb178-5G10) inhibited surface ATP generation and inhibited proliferation of HUVECs and MDA-MB-231 cells, but was not toxic by itself [69]. The same antibody induced apoptosis in A549 cells, accompanied by falls in extracellular ATP, intracellular pH, and ERK and AKT phosphorylation [14]. Another monoclonal antibody against ATP5B (mAb6F2C4) inhibited extracellular ATP synthesis, proliferation, anchorage-independent colony formation of the hepatoma cell line SMMC-7721 [65]; this antibody was also able to reduce hepatoma xenograft growth in vivo. The kringle 1–5 domain of plasminogen, an ecto-ATP synthase ligand, triggered caspase-dependent apoptosis in endothelial cells [52]. On the other hand, binding of apolipoprotein A1 to ecto-ATP synthase promoted the survival and differentiation of endothelial progenitor cells [51]. Differences in binding sites, effects on enzyme conformation, and protein interactions of ATP synthase ligands could explain these divergent effects. Additional microenvironmental factors, including acidic extracellular pH, may permit tumor-selective killing [13, 65]. Further studies will be required to elucidate the specific mechanisms of Apt63-induced programmed cell death in breast and prostate cancer, including effects on extracellular pH, reactive oxygen species, and purinergic nucleotides.

The quantitative relationship between ATP5B gene expression and surface ATP synthase is undetermined and likely complex: ATP synthase subunits are encoded by both nuclear and mitochondrial genomes, and are coordinately regulated through incompletely defined translational and post-translational means [38, 39, 41, 70–72]. Nonetheless, the associations we have identified between ATP5B gene expression and both metastasis-free and overall survival in breast and prostate cancer are remarkable. It is possible that proteomic analysis would demonstrate still stronger links. Comparing the proteomes of MCF-7 breast cancer and a highly invasive subclone, Pan et al. [42] found that another ATP synthase subunit, ATP5A was overexpressed in the aggressive subclone. ATP5A was identified on the surface of these cells, as well as on MDA-MB-231 and MDA-MB-453 breast cancer cell lines, but not on parental MCF-7 cells, or on non-tumorigenic MCF-10F breast epithelial cells [42]. In parallel, increased immunoreactive ATP5A was seen in 94% of breast cancers, as well as in 21.2% of normal tissues. This analysis did not discriminate between membrane and cytosolic staining, but the findings are consistent with a relationship between ATP5 protein levels and appearance on the plasma membrane. Other investigators have shown that ATP5B and other subunits of ATP synthase travel on lipid rafts that may shuttle between mitochondrial and plasma membranes [16, 73]; co-localization with caveolin-1 may be required to maintain a functional surface complex in vascular endothelium [73]. It will be important to examine the extent to which ATP5B expression correlates with surface ATP5B in future clinical studies of breast cancer prognosis.

A challenge in determining whether and how cancer cells utilize ecto-ATP synthase lies in the essential role of this enzyme in normal cell metabolism, and the unclear pathway by which the complex arrives at the cell surface. Our aptamer represents a new tool that will assist in elucidating these questions. Its rapid and selective cytotoxicity to cells expressing ecto-ATP5B may help to resolve structural and mechanistic questions about the importance of this complex to cancer cell survival and metastasis. Ultimately, the ability of Apt63 ability to target this important but poorly understood tumor antigen in primary breast and prostate tumors may help both to predict and mitigate the risk of future metastasis.

## Electronic supplementary material

Below is the link to the electronic supplementary material.

Supplementary material 1 (PDF 29570 KB)

Supplementary material 2 (MP4 5216 KB)
