# Supplementary material for: A novel RNA aptamer identifies plasma membrane ATP synthase beta subunit as an early marker and therapeutic target in aggressive cancer
Source: Breast Cancer Res Treat. 2019 Apr 20;176(2):271–89. doi: 10.1007/s10549-019-05174-3 (PMC6555781; doi:10.1007/s10549-019-05174-3)

Novel RNA aptamer identifies plasma membrane ATP synthase beta subunit as an early marker and therapeutic target in aggressive cancer

Speransky S[1] , Serafini P[2], Caroli J[3], Bicciato S[3], Lippman ME[1,4], Bishopric NH[1,4]

[1] Departments of Medicine and [2] Microbiology & Immunology, Sylvester Comprehensive Cancer Center, University of Miami Miller School of Medicine, Miami, USA
[3] Center for Genome Research, Department of Life Sciences, University of Modena and Reggio Emilia, Modena, Italy
[4] Department of Oncology, Georgetown Lombardi Comprehensive Cancer Center, Georgetown University, Washington DC, USA

Corresponding Author
Nanette H. Bishopric. MD
Email: nhb20@georgetown.edu

Supplemental Materials Table of Contents:

page
Online Resource 1..1
Online Resource 2 (Supplemental Methods)..................2-3
Online Resource 3..4-10
Online Resource 4..11
Online Resource 5..11

Online Resource 1. cDNA oligomer sequences of 5 aptamer candidates used for T7 in vitro transcription

aptamer	oligomer sequence
Apt413	5'-GGG GGA ATT CTA ATA CGA CTC ACT ATA GGG AGG ACG ATG CGG ATT ACC AAC TCG AAC GCC GAG AGTGTG GTC ACG TGT TCT GCA GAC GAC TCG CTG AGG ATC CGA GA-3'
Apt63	5'-GGG GGA ATT CTA ATA CGA CTC ACT ATA GGG AGG ACG ATG CGG GGA AGC AAC ACT TAG TCG CGA TTG ATA CGT GCG CAG TCA TCA GAC GAC TCG CTG AGG ATC CGA GA-3'
Apt 41	5'-GGG GGA ATT CTA ATA CGA CTC ACT ATA GGG AGG ACG ATG CGG ACG GAG GAT AGT TGC TAA TCG AGC GCT GCC GAC GCT CCA GAC GAC TCG CTG AGG ATC CGA GA-3'
Apt 132	5'-GGG GGA ATT CTA ATA CGA CTC ACT ATA GGG AGG ACG ATG CGG TCT CGA CTT TCC ACA CGC GTG TTC TTCGTC TGA TCT GGC CCA GAC GAC TCG CTG AGG ATC CGA GA-3'
Apt 521	5'-GGG GGA ATT CTA ATA CGA CTC ACT ATA GGG AGG ACG ATG CGG CCC ATC ACT CCC GCG TAT TGC GAA CGC ATC GTT ATT TAG CCA GAC GAC TCG CTG AGG ATC CGA GA-3'
Scr	5'-GGG GGA ATT CTA ATA CGA CTC ACT ATA GGG AGG ACG ATG CGG GGA AGC AAC ACT TAG TCG GTT AAA GCA ACA CTT AGC CAC CCA GAC GAC TCG CTG AGG ATC CGA GA-3'

Online Resource 2 (Supplemental Methods)

In vitro aptamer binding affinity and cytotoxicity assays

Two independent assays were carried out to evaluate Apt63 cytotoxicity in vitro: **(1)** direct visualization of Apt 63 cytotoxicity was performed in the IncuCyte® S3 Live-Cell Analysis System. LN3 and Pro 5 cells were seeded at 1×10^3 cells per well in 24-well, black-walled, clear-bottomed tissue culture plates (Corning, Corning, NY) in 300µl complete growth medium. Cells were allowed to grow for 24 hours. Cells were than treated with 1 nM of unlabeled Apt63, AptScr and 5 nM of SYTOX™ Green. The time-lapse video was recorded every 5 min for 1h 40min post aptamer treatment. **(2)** Apt63 cytotoxicity was evaluated by SYTOX™ Green uptake. PC3, PC3ML, RWPE, MDA-MB-23, MDA-MB-436, MCF7, MCF10A, T41, NR 67, LN3, Pro5 cell lines were seeded in 96-well, black-walled, clear-bottomed tissue culture plates (Corning, Corning, NY) in 100 µl complete growth medium. The number of seeded cells per well was experimentally determined for each cell line to be 60-70% confluent in 24 hours for all tested cultures. Cells were treated with 1 nM of unlabeled Apt63 and 5nM of SYTOX™ Green and endpoint readings were performed at 3 hours post aptamer treatment by recording SYTOX™ Green fluorescence. Readings were normalized to untreated cells and plotted using Excel.
Binding affinity of Apt63 to its membrane target was measured using the CellTiter-Glo® luminescent assay (Promega, Madison, WI). LN3 cells were seeded at 4×10^3 cells per well in 96-well, black-walled, clear- bottomed tissue culture plates (Corning, Corning, NY) in 100 µl complete growth medium and allowed to grow for 24 hours. For the experiment cells were treated in triplicate with unlabeled Apt63 at different concentrations (0.35 nM, 0.7 nM, 1nM, 1.4 nM, 2.3 nM, 7.0 nM, 10 nM, 15 nM). CellTiter-Glo® reagent was added to the wells at 1:1 vol/vol ratio as recommended by manufacturer and endpoint readings were performed at 60 min post aptamer treatment. The raw relative luminescence units (RLUs) from the plate reader were normalized to nontreated cells and plotted using GraphPad Prism 8 Software.
Both, luminescent and fluorescent readings were obtained using EnVision™ (PerkinElmer, USA) plate reader.

Mouse xenograft models and aptamer cytotoxicity in vivo

For visualization of Apt63 uptake and retain by xenograft tumor 14 male NOD.CB17-Prkdcscid/J mice (10 weeks old) were injected orthotopically into the right anterior lobe of the prostate with 2×10^6 LNCapLN3 and 2×10^6 LNCapPro5 cells. Each mouse received one type of cells. When xenograft tumors reach a palpable/visual size, each mouse received single tail vein injection with 1nmol of Alexa Fluor™ 647 labeled Apt63 and AptScr in 200 µl of PBS, 3 mice per each treatment. One mouse was injected with PBS only to establish auto fluorescence baseline. Live imaging of Alexa Fluor™ 647 labeled Apt63, and AptScr uptake was recorded in vivo on Xenogen IVIS-200 imaging system immediately after completed injections of all mice in the groups. Mice were re-imaged 3 hours post injection to record retention of labeled Apt63 and Apt Scr. Mouse IVIS imaging was performed by IUCAC approved standard procedure, were each mouse anesthetized by vaporized 2% isoflurane during imaging. After first imaging session mice were returned into resting cage and re-anesthetized for re-imaging. Mice were sacrificed after the last imaging session; xenograft tumors were recovered, frozen, sectioned, fixed and counterstained with DAPI as described in the following section. Sections were imaged on the confocal microscope to confirm aptamer retention by LN3 primary tumor xenografts.
For in vivo experiments to determine retention of Apt63 by mammary xenograft tumors, 21 NOD.CB17-Prkdcscid/J female mice (6-8 weeks old) were injected orthotopically into the mammary fat pad with 10^6 of MDA-MB-231 cells. When xenograft tumors reach a palpable/visual size, each mouse received single tail vein injection or 1 nmol of Alexa Fluor™ 647 labeled Apt63 and AptScr in 200 µl PBS, 9 mice with Apt63, 9 mice with AptScr. 3 mice were injected with PBS only for control. Mice were sacrificed at 6, 24 and 48 hours post injection. Control mice were sacrificed at one time point, 48 hours post injection. Xenograft tumors were recovered, frozen, processed for histology and immunofluorescent analysis as described in the following section. Small tumor samples were separated prior to fixation for DNA extraction.

Online Resource 2 continued (Supplemental Methods)

Tumor tissue and FFPE human biopsy arrays fluorescent staining and analysis

Xenograft tumors were removed from sacrificed mouse, oriented in cryomold with Tissue-Tek® O.C.T. Compound (Sakura® Finetek, USA) positioned flat on dry ice, and left to freeze. Frozen tissue blocks were sectioned on a Leica Cryomicrotome, -20C$^{0.}$ 10 μm thick sections were attached to glass slides and kept in -20 C^0 until being processed for staining. For Cy3-Apt63 staining, slides with frozen sections were removed from the freezer and placed immediately into 2% paraformaldehyde in PBS and fixed for 10 min. Next, slides were washed with PBS (3 times 1 min each). To block nonspecific RNA and DNA signals, sections were incubated 30 min in 10% dextran sulfate (Sigma) in PBS. Next, sections were washed (3 times 1 min each) and incubated with 1nM of Cy3-Apt63 for 30 min in the humidity chamber. After incubation, sections were briefly washed with PBS (3 times 1 min each) and counterstained with DAPI (Sigma) at 1ug final concentration for 5 minutes. For Alexa Fluor® 647 labeled Apt63 uptake study xenograft tumors were removed, frozen, sectioned, counterstained with DAPI (Sigma), 1ug final concentration for 5 minutes, washed and mounted with ProLong®Gold antifade reagent (Life Technologies).

Prostate and breast tissue biopsies microarrays were purchased from US Biomax, Inc (Rockville, MD). List of microarrays used in this study summarized in Online Resource 3. Prior staining with Cy3-Apt63, sections were deparaffinized by standard histological procedure and heated in 1X Citrate Buffer, pH 6.0 (Life Technologies, USA) for 30 minutes for antigen retrieval in the microwave. Next, sections were thoroughly washed with PBS (3 times 10 min each), incubated 30 min in 10% dextran sulfate (Sigma) in PBS to block nonspecific RNA and DNA signals, and incubated with 1nM of Cy3–Apt63 in PBS or 30 min in a humidified chamber. After incubation, sections were washed with PBS (3 times 5 min each), counterstained with DAPI (Sigma), 1ug final concentration for 5 minutes, washed and mounted with ProLong®Gold antifade reagent (Life Technologies).

For co-localization of Alexa Fluor® 647 ATP5B antibodies (ab223436, ABCAM) and Cy3-Apt63, the tissue arrays were processed for antigen retrieval as described above than stained with 1:200 diluted ATP5B antibodies for 1 hour in RT. Next, sections were thoroughly washed with PBS (3 times 10 min each) and stained with 1 nM of Cy3-Apt63, than washed, counterstained with DAPI and mounted. To confirm the specificity of Apt63 and ATP5B co-localization, staining was also performed in reverse, when sections were first stained with Cy3-Apt63, than with ATP5B antibodies, counterstained with DAPI and mounted.

Primary rabbit polyclonal EndoG antibodies (ab9647, ABCAM) were used on xenograft frozen sections to determine the localization of endonuclease G. Sections were incubated for 2 hours in RT with 1:500 dilution for primary antibodies and 1:500 for Alexa Fluor® 488 Goat anti-Rabbit secondary antibodies (A-11034, Thermo Fisher Scientific). Sections were counterstained with DAPI as described above and mounted.

Fluorescent images were obtained on a confocal microscope (Leica SP5) using 20x dry objective (Leica PL APO CS).

Online Resource 3. List of breast cancer and normal tissue biopsies. Pathology diagnosis, TNM, grade, stage, type of tumor were provided by Biomax, Inc. Apt63 score ("0" - no specific staining, "1" - yes specific staining) was assigned to each biopsy based on staining criteria listed in Methods. Total of 416 cases.

number	Organ	Pathology diagnosis	TNM	Grade	Stage	Type	aptamer staining 0/1	TMA ID number
1	Breast	Cancer adjacent normal breast tissue	-	-	-	NAT	0	BR723
2	Breast	Cancer adjacent normal breast tissue	-	-	-	NAT	0	BR723
3	Breast	Cancer adjacent normal breast tissue	-	-	-	NAT	0	BR723
4	Breast	Normal breast tissue (sparse)	-	-	-	normal	0	BR723
5	Breast	Normal breast duct tissue	-	-	-	normal	0	BR723
6	Breast	Adenosis (sparse)	-	-	-	benign	0	BR723
7	Breast	Adenosis	-	-	-	benign	0	BR723
8	Breast	Adenosis	-	-	-	benign	0	BR723
9	Breast	Adenosis (fibrofatty tissue)	-	-	-	benign	0	BR723
10	Breast	Adenosis	-	-	-	benign	0	BR723
11	Breast	Adenosis	-	-	-	benign	1	BR723
12	Breast	Adenosis	-	-	-	benign	0	BR723
13	Breast	Adenosis (fibrofatty tissue)	-	-	-	benign	0	BR723
14	Breast	Adenosis	-	-	-	benign	0	BR723
15	Breast	Fibroadenoma	-	-	-	benign	0	BR723
16	Breast	Fibroadenoma	-	-	-	benign	0	BR723
17	Breast	Fibroadenoma	-	-	-	benign	0	BR723
18	Breast	Fibroadenoma	-	-	-	benign	0	BR723
19	Breast	Hyperplasia of lobule	-	-	-	benign	0	BR723
20	Breast	Hyperplasia of lobule	-	-	-	benign	1	BR723
21	Breast	Hyperplasia of lobule	-	-	-	benign	0	BR723
22	Breast	Hyperplasia of lobule	-	-	-	benign	0	BR723
23	Breast	Adenosis with mild hyperplasia of ductal epithelium	-	-	-	benign	0	BR723
24	Breast	Hyperplasia (fibrofatty tissue)	-	-	-	benign	0	BR723
25	Breast	Mild hyperplasia of ductal epithelium	-	-	-	benign	0	BR723
26	Breast	Papillary hyperplasia of ductal epithelium	-	-	-	benign	1	BR723
27	Breast	Hyperplasia (fibrous tissue and blood vessel tissue)	-	-	-	benign	0	BR723
28	Breast	Mild atypical hyperplasia of lobular epithelium (sparse)	-	-	-	benign	0	BR723
29	Breast	Mild atypical hyperplasia of lobular epithelium	-	-	-	benign	0	BR723
30	Breast	Mild atypical hyperplasia of lobular epithelium (sparse)	-	-	-	benign	0	BR723
31	Breast	Mild atypical hyperplasia of ductal epithelium	-	-	-	benign	0	BR723
32	Breast	Mild atypical hyperplasia of lobular epithelium	-	-	-	benign	0	BR723
33	Breast	Moderate atypical hyperplasia of ductal epithelium	-	-	-	benign	0	BR723
34	Breast	Hyperplasia (fibrofatty tissue)	-	-	-	benign	0	BR723
35	Breast	Mild atypical hyperplasia of lobular epithelium	-	-	-	benign	0	BR723
36	Breast	Mild atypical hyperplasia of lobular epithelium	-	-	-	benign	1	BR723
37	Breast	Intraductal carcinoma	T1N0M0	-	1	malignant	0	BR723
38	Breast	Intraductal carcinoma	TisN0M0	-	0	malignant	0	BR723
39	Breast	Intraductal carcinoma	T2N0M0	-	2	malignant	0	BR723
40	Breast	Invasive ductal carcinoma	T2N0M0	1	2	malignant	0	BR723
41	Breast	Invasive ductal carcinoma	T4cN0M0	2	3	malignant	1	BR723
42	Breast	Invasive ductal carcinoma	T2N1M0	2	2	malignant	0	BR723
43	Breast	Invasive ductal carcinoma	T1N0M0	2	1	malignant	1	BR723
44	Breast	Invasive ductal carcinoma	T2N0M0	2	2	malignant	0	BR723
45	Breast	Invasive ductal carcinoma (adenosis)	T2N0M0	2	2	malignant	0	BR723
46	Breast	Invasive ductal carcinoma	T2N0M0	-	2	malignant	1	BR723
47	Breast	Invasive ductal carcinoma	T2N1M0	2	2	malignant	0	BR723
48	Breast	Invasive ductal carcinoma	T2N0M0	2	2	malignant	0	BR723
49	Breast	Invasive ductal carcinoma	T3N1M0	2	2	malignant	1	BR723
50	Breast	Invasive ductal carcinoma (sparse)	T2N0M0	-	2	malignant	0	BR723
51	Breast	Invasive ductal carcinoma	T3N1M1	1--2	4	malignant	0	BR723
52	Breast	Invasive ductal carcinoma	T2N0M0	2	2	malignant	1	BR723
53	Breast	Invasive ductal carcinoma	T2N0M0	3	2	malignant	1	BR723
54	Breast	Invasive ductal carcinoma	T2N0M0	3	2	malignant	1	BR723
55	Breast	Invasive ductal carcinoma	T4N1M0	3	3	malignant	1	BR723
56	Breast	Invasive ductal carcinoma	T2N0M0	3	2	malignant	0	BR723
57	Breast	Invasive ductal carcinoma	T2N0M0	3	2	malignant	1	BR723
58	Breast	Medullary carcinoma	T2N0M0	-	2	malignant	1	BR723

59	Breast	Medullary carcinoma	T1N0M0	-	1	malignant	0	BR723
60	Breast	Medullary carcinoma	T1N0M0	-	1	malignant	1	BR723
61	Breast	Mucinous carcinoma	T2N0M0	-	2	malignant	0	BR723
62	Breast	Mucinous carcinoma	T1N0M0	-	1	malignant	1	BR723
63	Breast	Invasive lobular carcinoma	T2N0M0	-	2	malignant	0	BR723
64	Breast	Invasive lobular carcinoma	T4N0M0	-	3	malignant	0	BR723
65	Breast	Invasive lobular carcinoma	T4N1M0	-	3	malignant	0	BR723
66	Breast	Metastatic invasive ductal carcinoma	-	-	-	MET.	1	BR723
67	Breast	Metastatic invasive ductal carcinoma	-	-	-	MET.	1	BR723
68	Breast	Metastatic invasive ductal carcinoma	-	-	-	MET.	1	BR723
69	Breast	Metastatic invasive ductal carcinoma	-	-	-	MET.	0	BR723
70	Breast	Metastatic invasive ductal carcinoma	-	-	-	MET.	1	BR723
71	Breast	Metastatic invasive ductal carcinoma	-	-	-	MET.	1	BR723
72	Breast	Invasive ductal carcinoma	T2N0M0	1	2	malignant	0	BR8017
73	Breast	Invasive ductal carcinoma	T3N0M0	1--2	2	malignant	1	BR8017
74	Breast	Invasive ductal carcinoma	T2N0M0	1	2	malignant	0	BR8017
75	Breast	Invasive ductal carcinoma	TisN0M0	1	0	malignant	1	BR8017
76	Breast	Invasive ductal carcinoma	T2N0M0	2	2	malignant	1	BR8017
77	Breast	Invasive ductal carcinoma	T2N0M0	2	2	malignant	0	BR8017
78	Breast	Invasive ductal carcinoma	T2N0M0	2	2	malignant	1	BR8017
79	Breast	Invasive ductal carcinoma	T4cN0M0	2	2	malignant	0	BR8017
80	Breast	Invasive ductal carcinoma	T2N0M0	2	2	malignant	0	BR8017
81	Breast	Invasive ductal carcinoma	T2N0M0	2	2	malignant	1	BR8017
82	Breast	Invasive ductal carcinoma	T2N0M0	2	2	malignant	0	BR8017
83	Breast	Invasive ductal carcinoma	T3N1M0	2	2	malignant	1	BR8017
84	Breast	Invasive ductal carcinoma	T2N0M0	2	2	malignant	1	BR8017
85	Breast	Invasive ductal carcinoma	T3N0M0	2	2	malignant	0	BR8017
86	Breast	Invasive ductal carcinoma	T3N0M0	2	2	malignant	1	BR8017
87	Breast	Invasive ductal carcinoma	T2N0M0	2	2	malignant	1	BR8017
88	Breast	Invasive ductal carcinoma	T2N0M0	2	2	malignant	0	BR8017
89	Breast	Invasive ductal carcinoma	T2N0M0	1	2	malignant	1	BR8017
90	Breast	Invasive ductal carcinoma	T3N1M0	2	2	malignant	0	BR8017
91	Breast	Invasive ductal carcinoma	T2N0M0	2	2	malignant	1	BR8017
92	Breast	Invasive ductal carcinoma	T4bN0M0	2	2	malignant	1	BR8017
93	Breast	Invasive ductal carcinoma	T2N0M0	2	2	malignant	1	BR8017
94	Breast	Invasive ductal carcinoma	T2N0M0	2	2	malignant	0	BR8017
95	Breast	Invasive ductal carcinoma	T2N0M0	2	2	malignant	0	BR8017
96	Breast	Invasive ductal carcinoma	T2N0M0	2	2	malignant	0	BR8017
97	Breast	Invasive ductal carcinoma	T3N0M0	2	2	malignant	1	BR8017
98	Breast	Invasive ductal carcinoma	T3N0M0	2	2	malignant	0	BR8017
99	Breast	Invasive ductal carcinoma	T2N0M0	2	2	malignant	1	BR8017
100	Breast	Invasive ductal carcinoma	T2N1M0	2	2	malignant	0	BR8017
101	Breast	Invasive ductal carcinoma	T2N1M0	2	2	malignant	1	BR8017
102	Breast	Invasive ductal carcinoma	T2N0M0	2	2	malignant	0	BR8017
103	Breast	Invasive ductal carcinoma	T3N0M0	2	2	malignant	0	BR8017
104	Breast	Invasive ductal carcinoma	T2N0M0	2	2	malignant	0	BR8017
105	Breast	Invasive ductal carcinoma	T2N0M0	2	2	malignant	1	BR8017
106	Breast	Invasive ductal carcinoma	T2N1M0	1	2	malignant	1	BR8017
107	Breast	Invasive ductal carcinoma	T2N0M0	2	2	malignant	1	BR8017
108	Breast	Invasive ductal carcinoma	T2N0M0	2	2	malignant	1	BR8017
109	Breast	Invasive ductal carcinoma	T2N0M0	2	2	malignant	1	BR8017
110	Breast	Invasive ductal carcinoma	T2N0M0	2	2	malignant	0	BR8017
111	Breast	Invasive ductal carcinoma	T2N0M0	1	2	malignant	1	BR8017
112	Breast	Invasive ductal carcinoma	T2N0M0	2	2	malignant	1	BR8017
113	Breast	Invasive ductal carcinoma	T2N0M0	2	2	malignant	1	BR8017
114	Breast	Invasive ductal carcinoma	T2N0M0	2	2	malignant	1	BR8017
115	Breast	Invasive ductal carcinoma	T2N0M0	2	2	malignant	0	BR8017
116	Breast	Invasive ductal carcinoma	T2N0M0	2	2	malignant	0	BR8017
117	Breast	Invasive ductal carcinoma	T2N0M0	2	2	malignant	0	BR8017
118	Breast	Invasive ductal carcinoma	T2N0M0	2	2	malignant	0	BR8017

119	Breast	Invasive ductal carcinoma	T3N0M0	2	2	malignant	1	BR8017
120	Breast	Invasive ductal carcinoma	T2N0M0	1	2	malignant	0	BR8017
121	Breast	Invasive ductal carcinoma	T2N0M0	2	2	malignant	0	BR8017
122	Breast	Invasive ductal carcinoma	T2N0M0	3	2	malignant	0	BR8017
123	Breast	Invasive ductal carcinoma	T2N1M0	3	2	malignant	1	BR8017
124	Breast	Invasive ductal carcinoma	T2N0M0	3	2	malignant	0	BR8017
125	Breast	Invasive ductal carcinoma	T2N0M0	3	2	malignant	0	BR8017
126	Breast	Invasive ductal carcinoma	T2N0M0	3	2	malignant	0	BR8017
127	Breast	Invasive ductal carcinoma	T2N0M0	3	2	malignant	1	BR8017
128	Breast	Invasive ductal carcinoma	T2N1M0	3	2	malignant	1	BR8017
129	Breast	Invasive ductal carcinoma	T2N0M0	3	2	malignant	0	BR8017
130	Breast	Invasive ductal carcinoma	T2N0M0	3	2	malignant	0	BR8017
131	Breast	Invasive ductal carcinoma	T2N0M0	2	2	malignant	0	BR8017
132	Breast	Invasive lobular carcinoma (sparse)	T2N0M0	-	2	malignant	0	BR8017
133	Breast	Invasive ductal carcinoma	T2N2M0	2	3	malignant	0	BR8017
134	Breast	Invasive ductal carcinoma	T3N2M0	3	3	malignant	0	BR8017
135	Breast	Invasive ductal carcinoma	T4N2M0	3	3	malignant	0	BR8017
136	Breast	Invasive ductal carcinoma	T4N1M0	3	3	malignant	1	BR8017
137	Breast	Invasive ductal carcinoma	T2N2M0	3	3	malignant	1	BR8017
138	Breast	Invasive ductal carcinoma	T4N1M0	3	3	malignant	0	BR8017
139	Breast	Invasive ductal carcinoma	T4N1M0	2	3	malignant	1	BR8017
140	Breast	Invasive ductal carcinoma	T2N2M0	2	3	malignant	1	BR8017
141	Breast	Invasive ductal carcinoma	T4N0M0	2	3	malignant	0	BR8017
142	Breast	Invasive ductal carcinoma	T4bN0M0	3	3	malignant	1	BR8017
143	Breast	Invasive ductal carcinoma	T4bN0M0	3	3	malignant	1	BR8017
144	Lymph node	Metastatic invasive ductal carcinoma	-	3	-	MET.	0	BR8017
145	Lymph node	Metastatic invasive ductal carcinoma	-	3	-	MET.	1	BR8017
146	Lymph node	Metastatic invasive ductal carcinoma	-	3	-	MET.	1	BR8017
147	Lymph node	Metastatic invasive ductal carcinoma	-	3	-	MET.	1	BR8017
148	Lymph node	Metastatic invasive ductal carcinoma	-	3	-	MET.	1	BR8017
149	Lymph node	Metastatic invasive ductal carcinoma	-	3	-	MET.	1	BR8017
150	Lymph node	Metastatic invasive ductal carcinoma	-	3	-	MET.	0	BR8017
151	Lymph node	Metastatic invasive ductal carcinoma	-	3	-	MET.	1	BR8017
152	Lymph node	Metastatic invasive ductal carcinoma from breast	-	2	-	Metastasis	1	BR8017
153	Lymph node	Metastatic invasive ductal carcinoma from breast	-	1	-	Metastasis	1	BR8017
154	Lymph node	Metastatic invasive ductal carcinoma from breast	-	2	-	Metastasis	1	BR8017
155	Lymph node	Metastatic invasive ductal carcinoma from breast	-	2	-	Metastasis	1	BR8017
156	Lymph node	Metastatic invasive ductal carcinoma from breast	-	2	-	Metastasis	1	BR8017
157	Lymph node	Metastatic invasive ductal carcinoma from breast	-	2	-	Metastasis	1	BR8017
158	Lymph node	Metastatic invasive ductal carcinoma from breast	-	2	-	Metastasis	1	BR8017
159	Lymph node	Metastatic invasive ductal carcinoma from breast	-	2	-	Metastasis	1	BR8017
160	Lymph node	Metastatic invasive ductal carcinoma from breast	-	2	-	Metastasis	1	BR8017
161	Lymph node	Metastatic invasive ductal carcinoma from breast	-	2	-	Metastasis	0	BR8017
162	Lymph node	Metastatic invasive ductal carcinoma from breast	-	2	-	Metastasis	1	BR8017
163	Lymph node	Metastatic invasive ductal carcinoma from breast	-	2	-	Metastasis	1	BR8017
164	Lymph node	Metastatic invasive ductal carcinoma from breast	-	2	-	Metastasis	1	BR8017
165	Lymph node	Metastatic invasive ductal carcinoma from breast	-	2	-	Metastasis	1	BR8017
166	Lymph node	Metastatic invasive ductal carcinoma from breast	-	2	-	Metastasis	1	BR8017
167	Lymph node	Metastatic invasive ductal carcinoma from breast	-	2	-	Metastasis	1	BR8017
168	Lymph node	Metastatic invasive ductal carcinoma from breast	-	2	-	Metastasis	1	BR8017
169	Lymph node	Metastatic invasive ductal carcinoma from breast	-	2	-	Metastasis	1	BR8017
170	Lymph node	Metastatic invasive ductal carcinoma from breast	-	2	-	Metastasis	1	BR8017
171	Lymph node	Metastatic invasive ductal carcinoma from breast	-	2	-	Metastasis	1	BR8017
172	Lymph node	Metastatic invasive ductal carcinoma from breast	-	2	-	Metastasis	1	BR8017
173	Lymph node	Metastatic invasive ductal carcinoma from breast	-	3	-	Metastasis	1	BR8017
174	Lymph node	Metastatic invasive ductal carcinoma from breast	-	2	-	Metastasis	0	BR8017
175	Lymph node	Metastatic invasive ductal carcinoma from breast	-	2	-	Metastasis	1	BR8017
176	Lymph node	Metastatic invasive ductal carcinoma from breast	-	2	-	Metastasis	1	BR8017
177	Lymph node	Metastatic invasive ductal carcinoma from breast	-	2	-	Metastasis	1	BR8017
178	Lymph node	Metastatic invasive ductal carcinoma from breast	-	2	-	Metastasis	1	BR8017

179	Lymph node	Metastatic invasive ductal carcinoma from breast	-	2	-	Metastasis	1	BR8017
180	Lymph node	Metastatic invasive ductal carcinoma from breast	-	2	-	Metastasis	1	BR8017
181	Lymph node	Metastatic invasive ductal carcinoma from breast	-	2	-	Metastasis	1	BR8017
182	Lymph node	Metastatic invasive ductal carcinoma from breast (fibrous	-	-	-	Metastasis	1	BR8017
183	Lymph node	Metastatic invasive ductal carcinoma from breast	-	2	-	Metastasis	1	BR8017
184	Breast	Invasive ductal carcinoma	T3N0M0	1	2	Malignant	1	BR2082b
185	Breast	Invasive ductal carcinoma	T1N0M0	1	1	Malignant	0	BR2082b
186	Breast	Invasive ductal carcinoma	T2N0M0	1	2	Malignant	0	BR2082b
187	Breast	Invasive ductal carcinoma	T2N0M0	2	2	Malignant	0	BR2082b
188	Breast	Invasive ductal carcinoma	T2N2M0	2	3	Malignant	1	BR2082b
189	Breast	Invasive ductal carcinoma	T2N0M0	1	2	Malignant	0	BR2082b
190	Breast	Invasive ductal carcinoma	T1CN0M0	2	1	Malignant	1	BR2082b
191	Breast	Invasive ductal carcinoma	T2N0M0	2	2	Malignant	1	BR2082b
192	Breast	Invasive ductal carcinoma	T4N0M0	2	3	Malignant	1	BR2082b
193	Breast	Invasive ductal carcinoma	T2N1M0	2	2	Malignant	0	BR2082b
194	Breast	Invasive ductal carcinoma of No. 44	T2N1M0	2	2	Malignant	0	BR2082b
195	Breast	Invasive ductal carcinoma	T1CN0M0	2	1	Malignant	0	BR2082b
196	Breast	Invasive ductal carcinoma	T3N2M0	2	3	Malignant	0	BR2082b
197	Breast	Invasive ductal carcinoma	T2N0M0	2	2	Malignant	0	BR2082b
198	Breast	Invasive ductal carcinoma	T2N0M0	2	2	Malignant	0	BR2082b
199	Breast	Invasive ductal carcinoma	T2N0M0	2	2	Malignant	1	BR2082b
200	Breast	Invasive ductal carcinoma	T2N0M0	2	2	Malignant	0	BR2082b
201	Breast	Invasive ductal carcinoma	T2N0M0	2	2	Malignant	0	BR2082b
202	Breast	Invasive ductal carcinoma	T2N0M0	2	2	Malignant	0	BR2082b
203	Breast	Invasive ductal carcinoma	T2N1M0	2	2	Malignant	1	BR2082b
204	Breast	Invasive ductal carcinoma	T4N2M0	2	3	Malignant	0	BR2082b
205	Breast	Invasive ductal carcinoma	T4BN0M0	2	3	Malignant	0	BR2082b
206	Breast	Invasive ductal carcinoma	T2N0M0	2	2	Malignant	0	BR2082b
207	Breast	Invasive ductal carcinoma	T1N0M0	2	1	Malignant	0	BR2082b
208	Breast	Invasive ductal carcinoma	T2N0M0	2	2	Malignant	0	BR2082b
209	Breast	Invasive ductal carcinoma	T4N0M0	2	3	Malignant	0	BR2082b
210	Breast	Invasive ductal carcinoma	T2N0M0	2	2	Malignant	0	BR2082b
211	Breast	Invasive ductal carcinoma	T2N0M0	2	2	Malignant	1	BR2082b
212	Breast	Invasive ductal carcinoma	T1N0M0	2	1	Malignant	1	BR2082b
213	Breast	Invasive ductal carcinoma	T2N0M1	2	2	Malignant	1	BR2082b
214	Breast	Invasive ductal carcinoma	T2N1M0	2	2	Malignant	1	BR2082b
215	Breast	Invasive ductal carcinoma	T2N0M0	2	2	Malignant	1	BR2082b
216	Breast	Invasive ductal carcinoma	T1N0M0	2	1	Malignant	1	BR2082b
217	Breast	Invasive ductal carcinoma	T1N2M0	2	3	Malignant	0	BR2082b
218	Breast	Invasive ductal carcinoma	T2N0M0	2	2	Malignant	0	BR2082b
219	Breast	Invasive ductal carcinoma (brease tissue)	T1N0M0	-	1	Malignant	1	BR2082b
220	Breast	Invasive ductal carcinoma	T2N0M0	2	2	Malignant	1	BR2082b
221	Breast	Invasive ductal carcinoma	T2N0M0	2	2	Malignant	1	BR2082b
222	Breast	Invasive ductal carcinoma (fatty tissue and blood vessel)	T2N2M0	-	3	Malignant	0	BR2082b
223	Breast	Invasive ductal carcinoma	T4N0M0	2	3	Malignant	0	BR2082b
224	Breast	Invasive ductal carcinoma	T2N0M0	2	2	Malignant	0	BR2082b
225	Breast	Invasive ductal carcinoma	T2N0M0	2	2	Malignant	1	BR2082b
226	Breast	Invasive ductal carcinoma	T2N0M0	2	2	Malignant	1	BR2082b
227	Breast	Invasive ductal carcinoma	T1N0M0	2	1	Malignant	1	BR2082b
228	Breast	Invasive ductal carcinoma	T1N1M0	2	2	Malignant	1	BR2082b
229	Breast	Invasive ductal carcinoma	T2N0M0	2	2	Malignant	1	BR2082b
230	Breast	Invasive ductal carcinoma	T2N0M0	3	2	Malignant	1	BR2082b
231	Breast	Invasive ductal carcinoma	T2N0M0	2	2	Malignant	0	BR2082b
232	Breast	Invasive ductal carcinoma	T2N2M0	2	3	Malignant	1	BR2082b
233	Breast	Invasive ductal carcinoma	T2N0M0	2	2	Malignant	0	BR2082b
234	Breast	Invasive ductal carcinoma	T2N0M0	2	2	Malignant	0	BR2082b
235	Breast	Invasive ductal carcinoma	T2N0M0	2	2	Malignant	0	BR2082b
236	Breast	Invasive ductal carcinoma	T2N1M0	2	2	Malignant	0	BR2082b
237	Breast	Invasive ductal carcinoma	T2N0M0	2	2	Malignant	1	BR2082b
238	Breast	Invasive ductal carcinoma	T1N0M0	2	1	Malignant	1	BR2082b

239	Breast	Invasive ductal carcinoma	T4N1M0	2	3	Malignant	1	BR2082b
240	Breast	Invasive ductal carcinoma	T2N1M0	2	2	Malignant	0	BR2082b
241	Breast	Invasive ductal carcinoma	T2N0M0	2	2	Malignant	0	BR2082b
242	Breast	Invasive ductal carcinoma	T2N0M0	2	2	Malignant	0	BR2082b
243	Breast	Invasive ductal carcinoma	T3N2M0	3	3	Malignant	0	BR2082b
244	Breast	Invasive ductal carcinoma	T2N0M0	2	2	Malignant	0	BR2082b
245	Breast	Invasive ductal carcinoma	T3N0M0	2	3	Malignant	0	BR2082b
246	Breast	Invasive ductal carcinoma	T2N1M0	3	2	Malignant	1	BR2082b
247	Breast	Invasive ductal carcinoma	T2N2M0	3	3	Malignant	1	BR2082b
248	Breast	Invasive ductal carcinoma	T2N1M0	3	2	Malignant	1	BR2082b
249	Breast	Invasive ductal carcinoma	T4N0M0	3	3	Malignant	0	BR2082b
250	Breast	Invasive lobular carcinoma	T2N0M0	-	2	Malignant	0	BR2082b
251	Breast	Invasive lobular carcinoma	T2N2M0	-	3	Malignant	0	BR2082b
252	Breast	Invasive lobular carcinoma	T2N1M0	-	2	Malignant	1	BR2082b
253	Breast	Invasive lobular carcinoma (sparse)	T2N1M0	-	2	Malignant	0	BR2082b
254	Breast	Invasive lobular carcinoma	T2N0M0	-	2	Malignant	1	BR2082b
255	Breast	Invasive lobular carcinoma	T2N1M0	-	2	Malignant	1	BR2082b
256	Breast	Invasive lobular carcinoma	T4N0M0	-	3	Malignant	1	BR2082b
257	Breast	Invasive lobular carcinoma	T4N0M0	-	3	Malignant	0	BR2082b
258	Breast	Invasive lobular carcinoma	T2N0M0	-	2	Malignant	1	BR2082b
259	Breast	Invasive lobular carcinoma	T2N1M0	-	2	Malignant	1	BR2082b
260	Breast	Invasive lobular carcinoma	T2N0M0	-	2	Malignant	1	BR2082b
261	Breast	Invasive lobular carcinoma	T4N0M0	-	3	Malignant	1	BR2082b
262	Breast	Invasive lobular carcinoma	T2N1M0	-	2	Malignant	0	BR2082b
263	Breast	Invasive lobular carcinoma	T2N1M0	-	2	Malignant	0	BR2082b
264	Breast	Invasive lobular carcinoma	T2N1M0	-	2	Malignant	0	BR2082b
265	Breast	Invasive lobular carcinoma (breast tissue)	T2N0M0	-	2	Malignant	0	BR2082b
266	Breast	Invasive lobular carcinoma (sparse)	T2N1M0	-	2	Malignant	0	BR2082b
267	Breast	Invasive lobular carcinoma (sparse)	T2N1M0	-	2	Malignant	1	BR2082b
268	Breast	Invasive lobular carcinoma	T2N1M0	-	2	Malignant	0	BR2082b
269	Breast	Invasive lobular carcinoma	T3N1M0	-	3	Malignant	0	BR2082b
270	Breast	Invasive lobular carcinoma	T2N0M0	-	2	Malignant	1	BR2082b
271	Breast	Invasive lobular carcinoma	T2N1M0	-	2	Malignant	1	BR2082b
272	Breast	Squamous cell carcinoma	T1N0M0	1--2	1	Malignant	1	BR2082b
273	Breast	Squamous cell carcinoma	T3N0M0	2	2	Malignant	0	BR2082b
274	Breast	Squamous cell carcinoma	T4N0M0	2	3	Malignant	0	BR2082b
275	Breast	Squamous cell carcinoma	T1N0M0	2	1	Malignant	0	BR2082b
276	Breast	Intraductal carcinoma	TisN0M0	-	0	Malignant	0	BR2082b
277	Breast	Intraductal carcinoma (sparse)	TisN0M0	-	0	Malignant	1	BR2082b
278	Breast	Intraductal carcinoma	TisN0M0	-	0	Malignant	1	BR2082b
279	Breast	Intraductal carcinoma	TisN0M0	-	0	Malignant	1	BR2082b
280	Breast	Intraductal carcinoma	TisN0M0	-	0	Malignant	1	BR2082b
281	Breast	Intraductal carcinoma	TisN0M0	-	0	Malignant	1	BR2082b
282	Breast	Intraductal carcinoma	TisN0M0	-	0	Malignant	1	BR2082b
283	Breast	Intraductal carcinoma	TisN0M0	-	0	Malignant	1	BR2082b
284	Breast	Intraductal carcinoma (adenosis)	TisN0M0	-	0	Malignant	0	BR2082b
285	Breast	Intraductal carcinoma	TisN0M0	-	0	Malignant	1	BR2082b
286	Breast	Intraductal carcinoma	T2N0M0	-	2	Malignant	0	BR2082b
287	Breast	Intraductal carcinoma (breast tissue)	TisN0M0	-	0	Malignant	0	BR2082b
288	Breast	Intraductal carcinoma with early infiltration	T2N0M0	-	2	Malignant	1	BR2082b
289	Breast	Intraductal carcinoma	TisN0M0	-	0	Malignant	1	BR2082b
290	Breast	Intraductal carcinoma	TisN0M0	-	0	Malignant	1	BR2082b
291	Breast	Intraductal carcinoma	TisN0M0	-	0	Malignant	0	BR2082b
292	Breast	Intraductal carcinoma	TisN0M0	-	0	Malignant	0	BR2082b
293	Breast	Intraductal carcinoma	TisN0M0	-	0	Malignant	0	BR2082b
294	Breast	Intraductal carcinoma (duct tissue)	TisN0M0	-	0	Malignant	0	BR2082b
295	Breast	Intraductal carcinoma	T2N1M0	-	2	Malignant	0	BR2082b
296	Breast	Intraductal carcinoma	TisN0M0	-	0	Malignant	0	BR2082b
297	Breast	Intraductal carcinoma	T2N0M0	-	2	Malignant	0	BR2082b
298	Breast	Lobular carcinoma in situ (adenosis)	TisN0M0	-	0	Malignant	0	BR2082b

299	Breast	Lobular carcinoma in situ	TisN0M0	-	0	Malignant	0	BR2082b
300	Breast	Lobular carcinoma in situ	TisN0M0	-	0	Malignant	0	BR2082b
301	Breast	Fibroadenoma	-	-	-	Benign	1	BR2082b
302	Breast	Fibroadenoma	-	-	-	Benign	1	BR2082b
303	Breast	Fibroadenoma	-	-	-	Benign	1	BR2082b
304	Breast	Fibroadenoma	-	-	-	Benign	0	BR2082b
305	Breast	Fibroadenoma	-	-	-	Benign	0	BR2082b
306	Breast	Fibroadenoma	-	-	-	Benign	0	BR2082b
307	Breast	Fibroadenoma	-	-	-	Benign	0	BR2082b
308	Breast	Fibroadenoma	-	-	-	Benign	0	BR2082b
309	Breast	Adenosis with hyperplasia of breast duct	-	-	-	Hyperplasia	1	BR2082b
310	Breast	Adenosis with hyperplasia of breast duct	-	-	-	Hyperplasia	0	BR2082b
311	Breast	Adenosis with hyperplasia of breast duct	-	-	-	Hyperplasia	1	BR2082b
312	Breast	Moderate hyperplasia of breast duct	-	-	-	Hyperplasia	1	BR2082b
313	Breast	Atypical hyperplasia in duct (grade II)	-	-	-	Hyperplasia	1	BR2082b
314	Breast	Adenosis with hyperplasia of breast duct	-	-	-	Hyperplasia	0	BR2082b
315	Breast	Adenosis with hyperplasia of breast duct	-	-	-	Hyperplasia	0	BR2082b
316	Breast	Hyperplasia of breast duct (hyalinosis of fibrous tissue)	-	-	-	Hyperplasia	0	BR2082b
317	Breast	Adenosis with moderate hyperplasia of duct	-	-	-	Hyperplasia	0	BR2082b
318	Breast	Hyperplasia (sparse breast tissue)	-	-	-	Hyperplasia	0	BR2082b
319	Breast	Hyperplasia (adenosis)	-	-	-	Hyperplasia	0	BR2082b
320	Breast	Hyperplasia (adenosis)	-	-	-	Hyperplasia	1	BR2082b
321	Breast	Hyperplasia (adenosis)	-	-	-	Hyperplasia	1	BR2082b
322	Breast	Hyperplasia (adenosis)	-	-	-	Hyperplasia	1	BR2082b
323	Breast	Hyperplasia (fibrous tissue and blood vessel)	-	-	-	Hyperplasia	0	BR2082b
324	Breast	Hyperplasia (adenosis)	-	-	-	Hyperplasia	0	BR2082b
325	Breast	Cancer adjacent normal breast tissue	-	-	-	NAT	1	BR2082b
326	Breast	Cancer adjacent normal breast tissue (fatty tissue and fibr	-	-	-	NAT	0	BR2082b
327	Breast	Cancer adjacent normal breast tissue (fatty tissue and fibr	-	-	-	NAT	0	BR2082b
328	Breast	Cancer adjacent normal breast tissue (ductal ectasia)	-	-	-	NAT	1	BR2082b
329	Breast	Cancer adjacent normal breast tissue	-	-	-	NAT	1	BR2082b
330	Breast	Cancer adjacent normal breast tissue	-	-	-	NAT	0	BR2082b
331	Breast	Cancer adjacent normal breast tissue (fibrous tissue)	-	-	-	NAT	0	BR2082b
332	Breast	Cancer adjacent normal breast tissue	-	-	-	NAT	0	BR2082b
333	Breast	Cancer adjacent normal breast tissue	-	-	-	NAT	0	BR2082b
334	Breast	Cancer adjacent normal breast tissue with ductal epithelia	-	-	-	Normal	0	BR2082b
335	Breast	Cancer adjacent normal breast tissue	-	-	-	NAT	0	BR2082b
336	Breast	Cancer adjacent normal breast tissue (fibrous tissue and b	-	-	-	NAT	0	BR2082b
337	Breast	Cancer adjacent normal breast tissue (fibrous tissue and b	-	-	-	NAT	0	BR2082b
338	Breast	Cancer adjacent normal breast tissue of No. 9	-	-	-	NAT	0	BR2082b
339	Breast	Cancer adjacent normal breast tissue	-	-	-	NAT	0	BR2082b
340	Breast	Cancer adjacent normal breast tissue	-	-	-	NAT	1	BR2082b
341	Breast	Breast Breast invasive ductal carcinoma	T2N0M0	1	2	malignant	0	BR8013
342	Breast	Breast invasive ductal carcinoma	T2N0M0	1	2	malignant	0	BR8013
343	Breast	Breast invasive ductal carcinoma	T1N1M0	2	2	malignant	1	BR8013
344	Breast	Breast invasive ductal carcinoma	T2N0M0	2	2	malignant	1	BR8013
345	Breast	Breast invasive ductal carcinoma	T1N1M0	2	2	malignant	1	BR8013
346	Breast	Breast invasive ductal carcinoma	T2N0M0	2	2	malignant	1	BR8013
347	Breast	Breast invasive ductal carcinoma	T1N1M0	2	2	malignant	0	BR8013
348	Breast	Breast invasive ductal carcinoma	T2N0M0	2	2	malignant	0	BR8013
349	Breast	Breast invasive ductal carcinoma (fibrofatty tissue)	T2N0M0	-	2	malignant	1	BR8013
350	Breast	Breast invasive ductal carcinoma	T2N0M0	2	2	malignant	1	BR8013
351	Breast	Breast invasive ductal carcinoma	T2N0M0	3	2	malignant	1	BR8013
352	Breast	Breast invasive ductal carcinoma	T3N1M0	2	2	malignant	0	BR8013
353	Breast	Breast invasive ductal carcinoma	T2N0M0	2	2	malignant	1	BR8013
354	Breast	Breast invasive ductal carcinoma	T2N0M0	2	2	malignant	0	BR8013
355	Breast	Breast invasive ductal carcinoma (breast tissue)	T2N1M0	-	2	malignant	1	BR8013
356	Breast	Breast invasive ductal carcinoma	T2N1M0	2	2	malignant	0	BR8013
357	Breast	Breast invasive ductal carcinoma	T2N1M0	2	2	malignant	1	BR8013
358	Breast	Breast invasive ductal carcinoma	T4NIM0	2	3	malignant	0	BR8013

359	Breast	Breast invasive ductal carcinoma (hyperplasia of breast du	T2N1M0	-	2	malignant	1	BR8013
360	Breast	Breast invasive ductal carcinoma	T2N1M0	2	2	malignant	1	BR8013
361	Breast	Breast invasive ductal carcinoma	T2N1M0	2	2	malignant	0	BR8013
362	Breast	Breast invasive ductal carcinoma	T4NIM0	2	3	malignant	1	BR8013
363	Breast	Breast invasive ductal carcinoma	T2N1M0	2	2	malignant	1	BR8013
364	Breast	Breast invasive ductal carcinoma	T2N1M0	2	2	malignant	0	BR8013
365	Breast	Breast invasive ductal carcinoma	T2N1M0	2	2	malignant	0	BR8013
366	Breast	Breast invasive ductal carcinoma	T2N1M0	2	2	malignant	0	BR8013
367	Breast	Breast invasive ductal carcinoma	T2N1M0	2	2	malignant	0	BR8013
368	Breast	Breast invasive ductal carcinoma	T3N1M0	2	3	malignant	1	BR8013
369	Breast	Breast invasive ductal carcinoma	T2N1M0	2	2	malignant	0	BR8013
370	Breast	Breast invasive ductal carcinoma	T2N1M0	2	3	malignant	1	BR8013
371	Breast	Breast invasive ductal carcinoma	T4N2M0	2	3	malignant	1	BR8013
372	Breast	Breast invasive ductal carcinoma	T2N1M0	2	2	malignant	0	BR8013
373	Breast	Breast invasive ductal carcinoma	T2N1M0	2	2	malignant	0	BR8013
374	Breast	Breast invasive ductal carcinoma	T2N2M0	2	3	malignant	0	BR8013
375	Breast	Breast invasive ductal carcinoma	T2N1M0	2	2	malignant	0	BR8013
376	Breast	Breast invasive ductal carcinoma	T2N1M0	2	2	malignant	1	BR8013
377	Breast	Breast invasive ductal carcinoma	T2N1M0	2	2	malignant	0	BR8013
378	Breast	Breast invasive ductal carcinoma	T2N1M0	2	2	malignant	1	BR8013
379	Breast	Breast invasive ductal carcinoma	T2N1M0	2	2	malignant	1	BR8013
380	Breast	Breast invasive ductal carcinoma	T2N1M0	2	2	malignant	0	BR8013
381	Breast	Breast invasive ductal carcinoma	T2N1M0	2	2	malignant	1	BR8013
382	Breast	Breast invasive ductal carcinoma	T4N2M0	2	3	malignant	1	BR8013
383	Breast	Breast invasive ductal carcinoma	T3N2M0	2	3	malignant	1	BR8013
384	Breast	Breast invasive ductal carcinoma	T4N1M0	2	3	malignant	1	BR8013
385	Breast	Breast invasive ductal carcinoma	T2N1M0	2	2	malignant	1	BR8013
386	Breast	Breast invasive ductal carcinoma	T4N2M1	2	4	malignant	0	BR8013
387	Breast	Breast invasive ductal carcinoma (breast tissue)	T4N0M0	-	3	malignant	1	BR8013
388	Breast	Breast invasive ductal carcinoma (sparse)	T2N1M0	-	2	malignant	0	BR8013
389	Breast	Breast invasive ductal carcinoma	T2N1M0	1--2	2	malignant	1	BR8013
390	Breast	Breast invasive ductal carcinoma	T2N1M0	3	2	malignant	1	BR8013
391	Breast	Breast invasive ductal carcinoma	T2N1M0	3	2	malignant	1	BR8013
392	Breast	Breast invasive ductal carcinoma	T2N2M0	3	2	malignant	1	BR8013
393	Breast	Breast invasive ductal carcinoma	T2N1M0	2	2	malignant	0	BR8013
394	Breast	Breast invasive ductal carcinoma	T2N2M0	2	3	malignant	1	BR8013
395	Breast	Breast invasive ductal carcinoma	T2N1M0	2	2	malignant	1	BR8013
396	Breast	Breast invasive ductal carcinoma	T3N1M0	3	3	malignant	1	BR8013
397	Breast	Breast invasive ductal carcinoma	T2N1M0	2	2	malignant	1	BR8013
398	Breast	Breast invasive ductal carcinoma	T2N1M0	3	2	malignant	0	BR8013
399	Breast	Breast invasive ductal carcinoma	T3N1M0	3	3	malignant	1	BR8013
400	Breast	Breast invasive ductal carcinoma	T2N1M1	3	2	malignant	0	BR8013
401	Breast	Breast invasive ductal carcinoma	T2N1M0	3	2	malignant	1	BR8013
402	Breast	Breast invasive lobular carcinoma	T2N1M0	-	2	malignant	0	BR8013
403	Breast	Breast invasive lobular carcinoma	T2N1M0	-	2	malignant	0	BR8013
404	Breast	Breast invasive lobular carcinoma	T3N1M0	-	3	malignant	0	BR8013
405	Breast	Breast invasive lobular carcinoma	T2N1M0	-	2	malignant	0	BR8013
406	Breast	Breast invasive lobular carcinoma	T2N1M0	-	2	malignant	0	BR8013
407	Breast	Normal breast tissue (fibrofatty tissue)	-	-	-	normal	0	BR8013
408	Breast	Normal breast tissue	-	-	-	normal	0	BR8013
409	Breast	Normal breast tissue (breast duct tissue)	-	-	-	normal	0	BR8013
410	Breast	Normal breast tissue	-	-	-	normal	0	BR8013
411	Breast	Normal breast tissue (breast duct tissue)	-	-	-	normal	0	BR8013
412	Breast	Normal breast tissue	-	-	-	normal	0	BR8013
413	Breast	Normal breast tissue	-	-	-	normal	0	BR8013
414	Breast	Normal breast tissue	-	-	-	normal	0	BR8013
415	Breast	Normal breast tissue	-	-	-	normal	0	BR8013
416	Breast	Normal breast tissue (sparse)	-	-	-	normal	0	BR8013

Online Resource 4. Aptamer #63 and #41 show high specificity to aggressive prostate LN3 cell line. LN3 (top) and Pro5 (bottom) cancer cell lines were incubated with 1nmol of representative Cy3-labeled aptamers from 5 sequenced families. Aggressive LN3 cell line show positive staining by Apt63 and 41; Pro5 cell line doesn't show positive staining. Original confocal imaging magnification: 20x

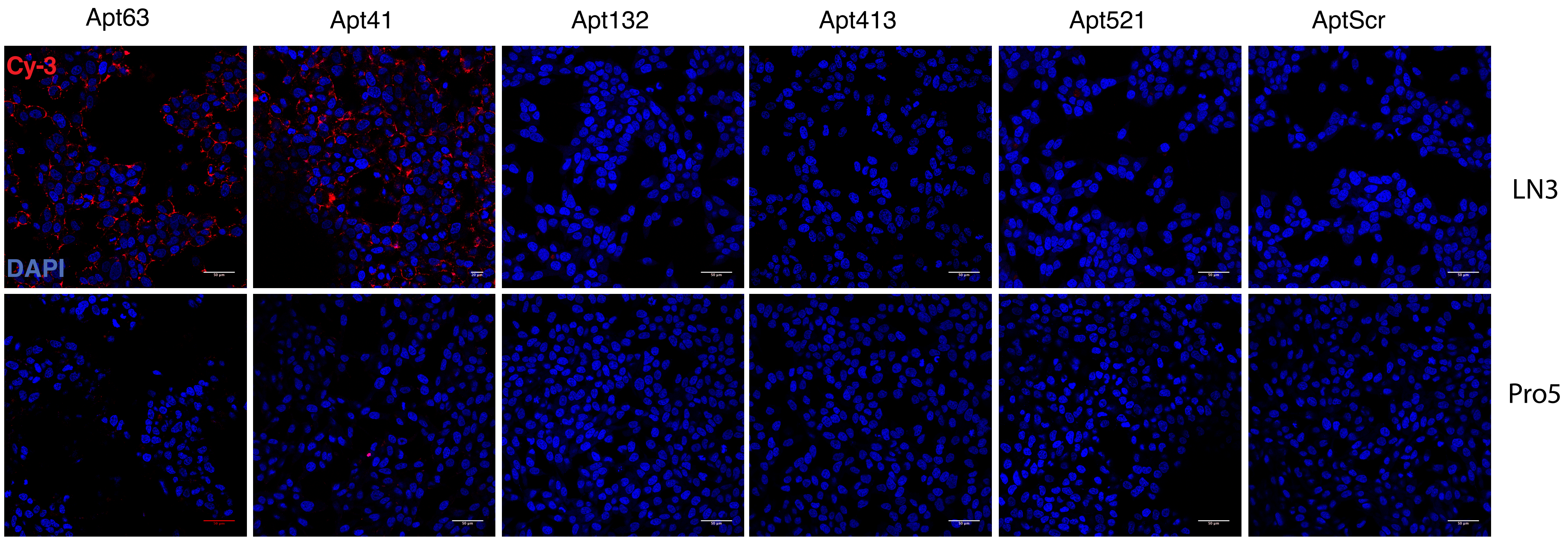

Online Resource 5. Apt63 efficiently discriminates between murine breast cancer cell lines with different metastatic potential, indicating inter-spe-cies conservation of the binding target. E0771.LMB and 4T1, highly metastatic cell lines show positive staining by Apt63; E0771 and 67 NR doesn't show positive staining. Original confocal imaging magnification: 20x

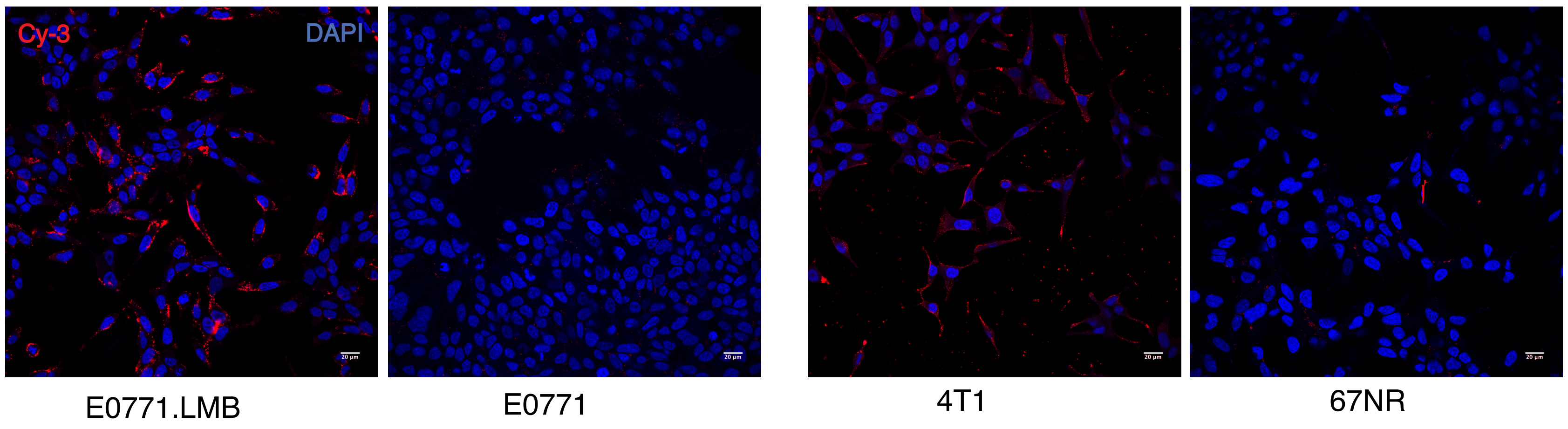

Supplement: Supplementary file 1 — Supplementary material 1 (PDF 29570 KB) [file 10549_2019_5174_MOESM1_ESM.pdf]
